# Industrial Microbial Technologies for Feed Protein Production from Non-Protein Nitrogen

**DOI:** 10.3390/microorganisms13040742

**Published:** 2025-03-25

**Authors:** Yuxin Ye, Yafan Cai, Fei Wang, Yi He, Yuxuan Yang, Zhengxiang Guo, Mengyu Liu, Huimin Ren, Shilei Wang, Dong Liu, Jingliang Xu, Zhi Wang

**Affiliations:** 1 School of Chemical Engineering, Zhengzhou University, Zhengzhou 450001, China; yeyuxin@gs.zzu.edu.cn (Y.Y.); caiyafan@zzu.edu.cn (Y.C.); wangf2022@gs.zzu.edu.cn (F.W.); yihe@gs.zzu.edu.cn (Y.H.); 17638192165@163.com (Y.Y.); gzx15238071996@163.com (Z.G.); liumengyu0037@163.com (M.L.); 17326266960@163.com (H.R.); shileiwang@zzu.edu.cn (S.W.); xujl@zzu.edu.cn (J.X.); 2 State Key Laboratory of Biobased Transport Fuel Technology, Zhengzhou University, Zhengzhou 450001, China; 3 College of Biotechnology and Pharmaceutical Engineering, Nanjing Tech University, Nanjing 211816, China; liudong@njtech.edu.cn

**Keywords:** industrial microbiology, non-protein nitrogen, feed protein, microbial protein, adaptive laboratory evolution

## Abstract

Due to the increasing global demand for feed protein, microbial protein has great potential of being able to feed sustainably. However, the application of microbial protein in the animal cultivation industry is still limited by its high cost and availability on scale. From the viewpoint of industrial production, it is vital to specify the crucial processes and components for further technical exploration and process optimization. This article presents state-of-the-art industrial microbial technologies for non-protein nitrogen (NPN) assimilation in feed protein production. Nitrogen sources are one of the main cost factors in the media used for large-scale microbial protein fermentation. Therefore, the available NPN sources for microbial protein synthesis, NPN utilization mechanisms, and fermentation technologies corresponding to the strain and NPN are reviewed in this paper. Especially, the random mutagenesis and adaptive laboratory evolution (ALE) approach combined with (ultra-) throughput screening provided the main impetus for strain evolution to increase the protein yield. Despite the underlying potential and technological advances in the production of microbial protein, extensive research and development efforts are still required before large-scale commercial application of microbial protein in animal feed.

## 1. Introduction

Protein, the most fundamental component of tissues, is essential for maintaining human health, and the current International Recommended Dietary Allowance (RDA) for protein is 0.8 g per kilogram of body weight [1]. Generally, plant-sourced proteins account for the vast majority (about 60%) of dietary protein, with the remainder coming from animal-sourced proteins [2]. As animal-sourced proteins have a much higher environmental impact (e.g., carbon footprint, arable land, and biodiversity loss) than plant-sourced proteins, plant-based proteins have become popular in the last decade for sustainability and ethical reasons [3,4]. However, animal-sourced protein is broadly recognized as having higher nutritional quality than plant-based protein, and the high consumption of animal-sourced proteins is expected to persist, especially in developed countries [5,6]. By 2050, the global population is expected to reach 8–10 billion, leading to a rapid increase in the consumption of animal protein [7,8]. The rising demand for livestock production is expected to increase the feed supply. By 2050, more than 1.1 billion tons of grains are projected to be used for animal feed, and monogastric animals are anticipated to consume the largest share, with a projected intake of 207 million tons of feed protein by 2030 [9]. Proteins are among the most expensive and limiting feed ingredients [10]. Although legume grains and some oilseed cakes are traditional protein sources for terrestrial monogastric animals (e.g., ruminants, pigs, and poultry), their long-term feeding is unsustainable [11]. Traditional livestock farming utilizes about 33% of terrestrial and 75% of freshwater resources, causing a serious problem of competition for food between humans and animals [12]. In addition, it is estimated that 57% of the greenhouse gas emissions from food production correspond to animal-based food production [13]. In summary, alternative protein sources for feed are necessary to substitute human-edible protein, satisfy the increasing need for feed protein driven by the global expansion of animal production, and reduce the environmental footprint of animal production.

In recent decades, alternative feed protein sources have been actively explored. Microbiol protein, obtained by culturing bacteria, yeast, fungi, or microalgae, has been recognized as a feasible and sustainable animal feed protein for more than four decades [10]. Microbial protein produced by different microbes has a high protein content (30%~70% of the dry weight of the cell) [14]. It is crucial to emphasize that the cultured microorganisms can be cultured on various agro-industrial processing wastes and cost-effective substrates, such as straw, dairy effluent, rice bran, biogas slurry, salad oil manufacturing wastewater, sugar beet pulp, and so on, which could not only lower the feed protein cost but also relieve environmental pollution [15,16]. Meanwhile, microbial fermentation can improve the protein quantity and quality, nutritional characteristics, digestibility, palatability, and safety of waste [17,18]. Especially, the fermentation of waste can be easily performed industrially at high intensity, and seasonal factors do not limit the effectiveness of the process.

To produce microbial protein, it is crucial to provide a substrate that contains nutrients accessible to microorganisms [19]. Particularly, carbon and nitrogen are the two most abundant nutrient elements for microorganisms to grow, and their metabolism is tightly coupled [20]. Various wastes are appealing substrates for microbial cell protein production because of their abundant carbohydrates [21]. However, most waste is composed of lignocellulosic materials, with cellulose as the major component, which microorganisms cannot directly utilize. Many studies have focused on the pretreatment of lignocellulosic materials, which can be converted to monosaccharides or disaccharides [22,23]. Recent reviews have reported advances in the utilization of carbon-abundant substrates by microorganisms for the production of microbial proteins and other chemicals [17,24,25].

To enhance the bioconversion efficiency of the microbial-producing substrate, a properly balanced metabolism of carbon and nitrogen is necessary for optimal microbial growth [26]. Generally, the carbon-to-nitrogen ratio (C/N) of agro-industrial waste is much higher than the optimum C/N ratio for microorganisms [27]. For example, the C/N ratio of bagasse is 58, while the optimum C/N ratio for the growth of *Methanosarcina* sp. is 25 [28]. In addition, most studies reported that the relative protein content of wastes could be improved by fermentation, which was mostly led by the decreasing amount of the dry weight, and the absolute protein content had no change [17,29]. Thus, additional nitrogen sources are usually required for microbial protein synthesis, and the selection of cost-effective nitrogen sources is vital for the industrial production of microbial protein. Recently, nitrogen wastes, such as wastewater, have triggered interest in their potential application in microbial protein production [30]. In addition, the metabolization of urea, ammonia solution, and ammonium sulfate by microorganisms has been studied in the fermentation process because of their lower cost compared to organic nitrogen sources (e.g., bone meal, fish meal, and soybean meal) [17,31]. The efficient utilization of low-cost carbon-abundant substrates and non-protein nitrogen (NPN, including atmospheric dinitrogen, urea, ammonia solution, and ammonium sulfate) by microorganisms could create microbial protein and improve the absolute protein content of the substrates, in addition to the transformation of organic nitrogen from the substrate to the microbial cell.

The future wide application of microbial protein in feed will depend heavily on improving bioconversion efficiency and reducing production costs using microbial fermentation technology. More detailed reviews of research on specific organisms have been summarized in previous studies [32,33,34]. This review focuses on recent advances in NPN sources and industrial fermentation technologies for microbial protein production. This review discusses the potential of NPN resources for industrial production, exploring in detail the feasibility of feed protein production, which has not been summarized previously. This review systematically examines the mechanisms of nitrogen assimilation, production methods, and scale-up perspectives of current industrial microbial technologies for producing feed proteins using NPN. This review highlights the central role of NPN utilization in reducing the cost of industrially produced microbial protein. Furthermore, the evolution and screening of industrial strains to enhance the bioconversion efficiency of NPN are discussed. This study highlights the future research focus and recommends pathways for the industrial production of feed protein from NPN.

## 2. Assimilation of Non-Protein Nitrogen Sources by Microorganisms

**Figure 1:**
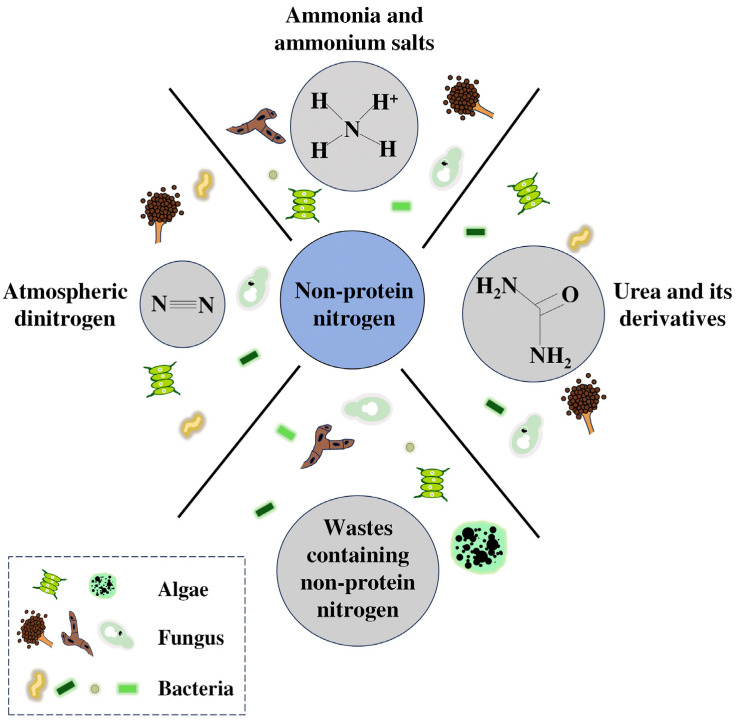
Assimilation of non-protein nitrogen sources by microorganisms.

Nitrogen is an essential component of microorganisms and a key media ingredient during fermentation, maintaining the physiological and biochemical activities of microbes [35,36]. Nitrogen assimilation by microorganisms plays a fundamental role in the synthesis of proteins, amino acids, nucleotides, and enzymes [37,38]. Together with the carbon source, the nitrogen source is one of the main cost factors in the media used for large-scale fermentation [39]. Nitrogen preferences are strain-dependent, and the utilization of different nitrogen sources can lead to various yields of microbial protein production. Generally, nitrogen sources for microorganisms can be classified into organic nitrogen sources and inorganic nitrogen sources. Organic nitrogen sources originate from plant and animal matter, including soybean, peanut, fish meal, cottonseed meal, corn pulp steep, and meat extract. Inorganic nitrogen sources are primarily obtained from inorganic sources, including atmospheric dinitrogen, ammonium hydroxide, and ammonium sulfate. Organic nitrogen refers to nitrogen in the form of proteins, peptides, and amino acids that can be directly fed to animals [40,41]. Thus, the transformation of organic nitrogen into microbial protein cannot alleviate the shortage of feed protein. Inorganic nitrogen, with the advantages of low cost and high yield, can serve as a nitrogen source for the production of microbial protein. In addition, urea, produced by the reaction of carbon dioxide with ammonia, can be rapidly hydrolyzed into ammonia by certain microorganisms [42]. Thus, inorganic nitrogen, urea, and its derivatives can be called NPN, which, together with wastes containing NPN or urea, can be used as nitrogen sources for feed protein production, in particular regarding the cost-effectiveness of NPN (Figure 1).

### 2.1. Atmospheric Dinitrogen

The Earth’s atmosphere comprises about 78% nitrogen, which occurs as dinitrogen gas (N_2_). Most microorganisms cannot utilize atmospheric dinitrogen directly, except for some nitrogen-fixing microorganisms that convert atmospheric dinitrogen into a form they can incorporate into their structure. Traditionally, nitrogen-fixing microorganisms were thought to include only prokaryotes; however, the discovery of *Candidatus* Atelocyanobacter thalassa (UCYN-A) has shown that some eukaryotes also possess nitrogen-fixing functions [43,44]. Biological nitrogen fixation can be categorized based on the symbiotic relationships between nitrogen-fixing microorganisms and host plants, including symbiotic, associative, and autotrophic nitrogen fixation [45]. Symbiotic nitrogen-fixing microorganisms must coexist with plants, while non-symbiotic nitrogen-fixing microorganisms (including associative nitrogen-fixing microorganisms and autotrophic nitrogen-fixing microorganisms) can survive independently [46]. Thus, non-symbiotic nitrogen-fixing microorganisms can be employed in industrial fermentation for microbial protein production. Non-symbiotic nitrogen-fixing microorganisms mainly include *Beijerinckia*, *Azotobacter*, *Azospirillum*, *Herbaspirillum*, *Gluconacetobacter*, *Burkholderia*, *Clostridium*, *Methanosarcina*, and *Paenibacillus* at the genus level [47]. Some non-symbiotic nitrogen-fixing microorganisms belong to a group known as nitrogen-fixing hydroxylating bacteria (such as *Xanthobacter*), which have been studied for their potential in protein production [48,49]. Compared to other non-symbiotic nitrogen-fixing microorganisms, nitrogen-fixing hydroxide bacteria offer a high microbial protein content (~70%) and can be produced in situ without ammonia outgassing [49]. Additionally, these bacteria have twice the energy conversion efficiency of soybean seeds in converting solar energy to biomass [50].

The nitrogen fixation efficiencies of the three different nitrogen-fixing microorganisms vary significantly [45]. Symbiotic nitrogen-fixing microorganisms can fix pure nitrogen ranging from 75 to 300 kg per hectare per year, while autochthonous nitrogen-fixing microorganisms can only fix approximately 20 kg of pure nitrogen per hectare per year in nature [51,52,53]. Nitrogenase plays a crucial role in the nitrogen fixation process of microorganisms [54]. However, nitrogenase is extremely sensitive to molecular oxygen, which becomes permanently deactivated in its presence; thus, nitrogen-fixing genes are only expressed at high levels under anaerobic conditions [55]. Additionally, nitrogen-fixing products of ammonia in the environment also inhibit the expression of nitrogen-fixing genes. The nitrogen fixation reaction is also a high-energy process because reducing one nitrogen molecule consumes eight high-energy electrons and 16 ATP [56,57,58]. Recently, genetic engineering has been conducted on nitrogen-fixing microorganisms to enhance their growth in industrial fermentation [59,60]. Disruption of signal transduction protein PII signaling and the expression of unidirectional adenylyltransferases can prevent nitrogen-fixing microorganisms from assimilating ammonia produced by nitrogen fixation through glutamine synthetase (GS) activation, resulting in a large exocytosis of ammonia and thereby reducing ammonia inhibition [61,62]. It was found that the expression of nitrogen fixation genes from *Azotobacter vinelandii* in a genetically engineered *Escherichia coli* could enhance the pentose phosphate pathway and upregulated genes related to the electron transport system to increase the efficiency of energy generation for nitrogen fixation [63]. Additionally, increased expression of genes associated with respiratory activity can remove oxygen to maintain the anaerobic environment required for nitrogenase activity [64]. However, the widespread expression of nitrogenase genes in the host has not yet been achieved because the regulatory coupling between host genes and heterologous nitrogen fixation genes is not yet fully understood [65]. To satisfy the high-energy demands of the nitrogen fixation process, cadmium sulfide nanocrystals were used to absorb light energy to drive nitrogenase fixation extracellularly [66]. In addition to the three key limiting factors mentioned above, other factors, such as the availability of electron mediators, pH reduction, and iron limitations, also influence nitrogenase activity [67,68]. To improve the efficiency of the nitrogen fixation process, it is essential to comprehensively consider the effects of various factors on nitrogenase activity.

### 2.2. Urea and Its Derivatives

Urea is a vital nitrogen fertilizer that serves as the “food of food” [69]. In mammals, urea, which is produced in the liver to detoxify ammonia, cannot be further metabolized [70]. Most microorganisms can secrete urease, converting urea back to ammonia for growth. Thus, in ruminant species, urea recycled from the liver can be metabolized by microbes in the rumen, providing a microbial protein source for milk or muscle protein synthesis. Moreover, considering the low price and high nitrogen content (46.7%) of urea, it has been used as NPN to partially substitute for feed protein in ruminant science in the early 20th century [71]. However, the amount of urea in the feed is strictly limited due to the ammonia toxicity caused by the discrepancy between the availability rate of urea-derived ammonia and the rate of nitrogen uptake by rumen bacteria [72]. Microbial protein produced using urea or its derivatives (e.g., biuret and urea phosphate) as a nitrogen source can be largely used to feed ruminants as well as monogastric animals [73,74]. Moreover, based on the nitrogen content, one gram of urea could be converted to 2.92 g of protein, which costs only 0.12 USD/kg (calculated based on the cost of urea, 0.35 USD/kg), which is much lower than the cost of protein from soybean, 1.24 USD/kg (calculated based on the cost of soybean, 0.57 USD/kg and 46% protein content) [75,76].

Urea is an interesting alternative nitrogen source for microbial fermentation, as it is more sustainable and cost calculations with a lower cost per mol nitrogen than that for ammonium sulfate. In addition, urea does not acidify the media; thus, less base addition is required for pH-controlled large-scale fermentation [77]. Hence, the effect of urea on the cell behavior and the production performance of microorganisms has been studied, which are diverse among the different strains, including prokaryotes, fungi, and microalgae [78,79]. Urea was found to be an alternative nitrogen source without a significant impact on the cell growth, transcriptome, and lipid production of three *Yarrowia lipolytica* strains compared to ammonium sulfate [80]. Similarly, urea and urine were found to be far more effective for biomass accumulation with higher growth rates and equivalent lipid production of the *Y. lipolytica* strain PO1f, compared to ammonium sulfate on an equivalent nitrogen basis, as reported by [81]. Urea also could be used as a sole nitrogen source for *Saccharomyces cerevisiae* growth, together with the liquefied corn starch solution as a carbon source [82]. The highest ethanol yield was achieved with 150 mM urea used, while a higher urea concentration inhibited growth. Similarly, it was found that the increase in urea concentration during *S. cerevisiae* strain cultivation could significantly decrease the number of viable cells, specific growth rate, and ethanol efficiency [83,84]. However, urea as a nitrogen source inhibited the growth of *Candida utilis* (12.7 g/L cell mass) compared to other organic sources (corn steep liquor, yeast extract, peptone, and soybean meal) and inorganic sources (ammonium sulfate and ammonium chloride) [85]. For microalgae, ammonium is generally described as a preferable nitrogen source because of its low uptake energy [86,87]. Recently, it was found that urea showed similar responses in biomass productivity when compared to nitrate or ammonium in the cultivation of *Chlamydomonas reinhardtii* and *Chlorella sorokiniana* species [88,89]. The growth rate of four microalgae species (*Chlorella vulgaris*, *Auxenochlorella protothecoides*, *C. sorokiniana*, and *Nannochloropsis oculate*) on various nitrogen sources, including urea, was determined, which confirmed that urea could be assimilated by the test microalgae with different growth rates [90]. The metabolism of urea in prokaryotes has been studied for many years and is related to the virulence of the pathogen or the conversion of urea in the host [91,92]. In particular, rumen bacteria can hydrolyze urea from feed to synthesize microbial protein [93]. However, few studies have reported the production of microbial protein in bacteria using urea. A lot of industrial prokaryotes, such as *Bacillus*, *Corynebacterium glutamicum*, and *Cyanobacteria* can metabolize urea [94,95,96,97].

Many microorganisms, including aerophilic, microaerophilic, and anaerobic strains, are capable of hydrolyzing urea for further nitrogen metabolization, which even comprises about 30% of the soil microbes [98]. Several studies have attempted to add urea to the medium for biofuel, bioethanol, biochemical, and biomass production, which could decrease the cost or enhance the yield [99,100,101]. However, the ability to assimilate urea varies among microbes. The amount of urea in the medium also affects the growth of the strain, and it is critical to explore the optimum amount of urea. It was found that a higher content of urea in the medium always leads to a decrease in the growth of the strain, which might be due to the rapid hydrolysis of urea and the lack of synchronization between nitrogen and the availability of carbohydrates [102]. For ruminants, slow-release urea products (dextrinized urea, coated urea, urea molasses licking bricks, etc.) have been designed for rumen feed to improve rumen fermentation and efficiency in urea use [103,104]. Thus, the batch addition of urea or controlled-release urea in microbial production may improve the amount of urea assimilated, especially during the saccharification and fermentation processes when lignocellulosic biomass is used as a substrate. Moreover, screening for urealytic bacteria is vital for the utilization of urea in industrial fermentation.

### 2.3. Ammonia and Ammonium Salts

Ammonia and ammonium salts are the preferred nitrogen sources for industrial fermentation due to their low cost. Ammonia is primarily produced using the Haber-Bosch process in industry, which involves the reaction of hydrogen with nitrogen [105]. Alternative methods, such as electrochemical synthesis, biomass utilization, and carbon capture, offer viable options for producing cost-effective green ammonia, resulting in reduced greenhouse gas emissions [106]. Additionally, ammonium salts are generated by reacting ammonia with hydrochloric acid, sulfuric acid, carbon dioxide, and other related compounds [107,108]. The annual international production of ammonia has reached 175 million tons [109]. Ammonia and ammonium salts can be utilized by many microorganisms, such as *Streptococcus*, *Succinivibrio*, *Clostridium sensu stricto*, *Oxalobacter*, *Bacteroidales*, *Candidatus scalindua*, *Nitrosopumilus* spp., and *Nitrospina* [110,111]. The microbial use of ammonium salts requires less energy than urea because ammonium salts can be assimilated as ammonium−nitrogen by most microorganisms [112].

In industrial fermentation, ammonia and ammonium salts are commonly added to fermentation media as nitrogen sources. Research has shown that the addition of ammonium sulfate can enhance the maximum specific growth rate from 0.24 h^−1^ to 0.27 h^−1^ of *S. cerevisiae* S101 [113]. In the later stages of fermentation, the addition of ammonium salts could promote the specific growth rate of *Aspergillus niger* PM1, with an increase of about twofold [114]. For bacteria, different species exhibit varying levels of resistance to ammonium salts. For instance, *C. glutamicum*, *E. coli*, and *Bacillus subtilis* are highly resistant and can thrive even at ammonium concentrations of 500 mM [115]. A concentration of 60 mM ammonium salts has been shown to enhance the growth of *Streptomyces fradiae* SF-2, resulting in approximately a 1-fold increase in the specific growth rate compared to conditions without ammonium salts [116]. However, *S. fradiae* is sensitive to ammonium, and its growth is inhibited by 20 mM ammonium [117]. Particularly, wastewater containing ammonium salts can be used as a nitrogen source by strains *Cupriavidus necator* H16 and *Xanthobacter viscosus* 7d, with CO_2_ as the carbon source to produce microbial protein feed, which could serve as a viable alternative to conventional feed sources like fishmeal or soybean protein [118]. Generally, microalgae can tolerate higher ammonium concentrations than other microorganisms [119]. For example, *C. vulgaris*, *Chlorella minutissima*, *C. reinhardtii,* and *Arthrospira platensis* can grow without inhibition in ammonium levels comparable with municipal wastewater [120]. Microalgae, such as *C. vulgaris* and *C. reinhardtii,* preferentially utilize ammonium for growth when both nitrate and ammonium are available [121]. Many researchers have focused on removing ammonium−nitrogen from wastewater using microalgae owing to their ammonia utilization ability [122,123].

In the medium, ammonia and ammonium salts dissociate into ammonium ions, which are taken up by microorganisms through membrane transporter proteins [124]. Ammonium ions react with glutamic acid to form glutamine with the aid of GS enzyme in the cell [125]. Glutamine could serve as a nitrogen donor for synthesizing amino acids, which are crucial for protein synthesis [126]. GS plays a vital role in converting inorganic ammonium ions to organic nitrogen. Both the species of microalgae and the concentration of ammonium had significant effects on GS activity [125,127]. Therefore, factors such as the strain species and the amount of ammonia and ammonium salts added must be considered to improve the ammonia−nitrogen conversion efficiency and protein yield in industrial production.

### 2.4. Wastes Containing Non-Protein Nitrogen

Nitrogen deposition and nitrogen-containing wastes are interconnected within the nitrogen cycle, and emissions from nitrogen-containing wastes worsen nitrogen deposition. The global average total nitrogen deposition flux to land areas in 2020 was 7.0 kg N/ha/yr, of which ammonium contributed 4.3 kg N/ha/yr [128]. Common nitrogenous wastes include municipal sludge, food waste, and livestock manure containing NPNs, such as urea and ammonium [129,130]. In addition, industrial and agricultural by-products (e.g., wastewater and digestate) also contain NPN, mainly in the form of ammonium−nitrogen (Table 1) [131].

Producing feed protein from nitrogen-rich waste streams is a promising alternative that could avoid all the inherent losses and pollution of NPN. Meanwhile, innocuous residues generated containing carbohydrates, lipids, proteins, vitamins, minerals, and bioactive compounds from industry could thus be used as substrates for the fermentation of microorganisms [143,144]. Industrial and agricultural by-products contain two nitrogen sources: organic nitrogen (proteins, peptides, and amino acids) and NPN. The fermentation of the former waste (containing organic nitrogen) could only improve the quality of protein, while the quantity of protein could not be increased. Producing feed protein from later waste can convert NPN to organic nitrogen. Feed protein can be produced not only from ammonium−nitrogen-rich biogas slurry but also from various nitrogenous wastes, including food, industrial and agricultural waste, and industrial by-products [145,146,147,148]. A mixture of various food wastes, such as fish waste, pineapple, banana, apple, and citrus peels, was fermented by *S. cerevisiae*, resulting in an increase in protein content from 8.52% to 40.19% [149]. Additionally, tofu waste can be fermented by *Chlorella* sp. with a protein content of 52.32% [150]. Industrial and agricultural wastes, such as sugarcane molasses, waste cooking oil, crude glycerol from biodiesel production, waste paper, lignin residues, methanol, and other industrial by-products, have been explored for producing feed protein [151,152,153]. For instance, the fermentation of *Y. lipolytica* in waste cooking oil produced 36.6 g/L of biomass with a protein content of 47% [154], and the fermentation of sugarcane molasses could produce 151.2 g/L of protein on a 10 L scale [147]. The fermentation of *Rhodotorula mucilaginosa* on wastepaper hydrolysate produced 2.1 g/L of protein on a 3 L scale [155].

This approach can add value to waste by allowing it to re-enter the feed protein production chain as an important step in the practice of the so-called circular economy and can also contribute to global food security. Thus, many companies are beginning to produce proteins from waste substrates [156]. Mycorena Company’s flagship product, Promyc, was produced through a process integrated into the circular economy. It uses by-products from industrial food production as raw materials and obtains Promyc via filamentous fungi fermentation, effectively transforming “waste” into “value”. Promyc can replace soybean meal, fish meal, and other feedstuffs for cattle, sheep, pigs, chickens, and other livestock. KnipBio Company produces KnipBio Meal (KBM), a single-cell protein for the aquaculture industry obtained from ethanol-fermented distillate concentrate, which offers a protein content comparable to that of fishmeal and is free of the anti-nutritional factors typically found in plant proteins. Nutrinsic Company utilizes bacterial fermentation of brewery wastewater to produce ProFloc^TM^ protein, which contains 60% protein and can serve as feed for fish and other animals. Saltgae Corporation has implemented a microalgae-based approach to treat industrial nitrogenous wastewater and synthesize protein for aquaculture through photosynthesis, using NPN as a nutrient source. The microalgae-based approach can recover 90% of the energy and nutrients in wastewater and reduce wastewater treatment costs by 59%. Furthermore, microalgae feed supplements contain high-value polyunsaturated fatty acids and a balanced amino acid profile, thus acting as a feasible substitute for fishmeal [157]. Unibio Company ferments methane with microorganisms to produce UniProtein R, which has a protein content of up to 71% and is approved for use in the animal and aquaculture industries [158].

It is important to mention that the waste must contain nutrients (particularly carbon and nitrogen) in a form that is accessible to the microorganism. In most agricultural waste, carbohydrates are present as cellulose and hemicellulose, which cannot be directly metabolized by most microorganisms. Many researchers have focused on developing chemical, physical, and/or biological techniques to deconstruct polysaccharides into monosaccharides to provide a carbon source for fermentation, as seen in recent reviews [159,160]. The form and content of NPN in waste are critical for the fermentation process. The NPN content in wastes like apple residues and banana peels is too low for effective microbial fermentation, requiring the addition of an external nitrogen source [161]. Certain NPNs that are tightly bound to organic matter in combined sewer overflow wastewater pose a challenge because they are difficult for microorganisms to utilize directly [162]. Additionally, it is important to consider the impact of waste on the fermentation process. For example, feed protein produced using wastewater containing harmful metals (such as copper, zinc, cadmium, chromium, mercury, iron, lead, nickel, and arsenic) is hazardous to animal health [163]. Some waste contains complex components, such as toxic organic matter, which poses a significant challenge to the growth of microorganisms [164]. For example, residual antimicrobials and antibiotics in pharmaceutical factory wastewater can disrupt the microbial ecology [165]. Many countries, such as the United States, Europe, and Japan, have already developed regulations concerning the production of feed proteins to ensure their quality and safety of the feed protein [166]. In the United States, agricultural waste used as a raw material for producing microbial proteins must meet the food safety and quality standards set by the Food and Drug Administration (FDA). In the European Union, the use of organic waste for producing microbial proteins must ensure that the waste source is safe and free from contamination by hazardous substances. Japan’s Ministry of Agriculture, Forestry, and Fisheries (MAFF) regulates the production of microbial proteins according to the Good Feed Manufacturing Practice (GFMP) to ensure compliance with Japanese food safety and quality standards.

## 3. Fundamental of Non-Protein Nitrogen Assimilation by Microorganisms

**Figure 2:**
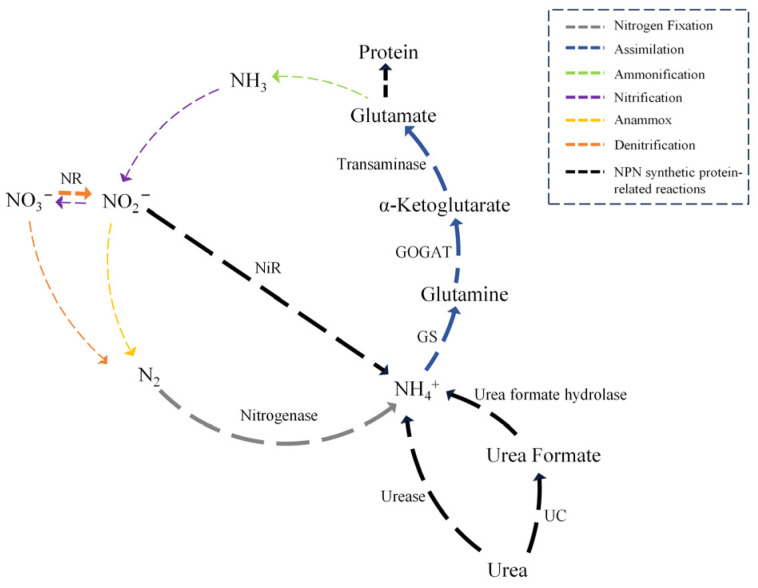
The representation of various NPN-synthesized feed proteins in the nitrogen cycle network. Ammonia in the nitrogen cycle pathway originates from the reduction and conversion of nitrate and nitrite, atmospheric dinitrogen capture, and urea decomposition. Protein synthesis requires ammonia to undergo a series of biochemical reactions, including converting ammonia to glutamine and α-ketoglutarate, and α-ketoglutarate to glutamate, which acts as an amino acid residue for protein synthesis. The color/thin line represents the nitrogen cycle network, and the black/thick line illustrates the conversion of NPN into feed protein. NR, nitrate reductase; NiR, nitrite reductase; GS, glutamine synthetase; GOGAT, glutamate synthetase; UC, urea carboxylase.

Nitrogen transformation in microorganisms is often described as a cycle consisting of six distinct processes: ammonification, nitrogen fixation, nitrification, denitrification, anaerobic ammonium oxidation (anammox), and assimilation [167]. In this nitrogen cycle network, atmospheric dinitrogen is converted to ammonia by nitrogen-fixing microorganisms, which is then converted to glutamate through assimilation. Microbial ammonification of organic nitrogen decomposes organic nitrogen to release ammonia molecules, which are then oxidized to nitrite and further to nitrate under the nitrification of nitrifying bacteria. Nitrate is then oxidized to atmospheric dinitrogen under anoxic conditions via denitrification, while nitrite is oxidized via anammox and released into the atmosphere. In this cycle, the conversion of NPN into feed proteins is represented by microbial assimilation (Figure 2). The role of NPN in microbial protein synthesis was demonstrated in the 1960s when Belasco and Henderickx showed the high efficiency of ammonium butanedioate in facilitating microbial protein synthesis. Further experiments by Zhu et al. demonstrated that organic ammonium salts can provide nitrogen for bacterial growth and cellulose digestion, while also promoting microbial protein synthesis [168]. A variety of microorganisms in nature can use NPN to synthesize proteins for their growth [169]. Microorganisms use enzymes to degrade NPN into ammonia, which is further assimilated into amino acids under the catalytic effect of enzymes and then metabolized by microorganisms to produce mycoprotein.

Nitrogenase, nitrite reductase (NiR), and glutamine synthetase (GS) are three key enzymes in the assimilation of NPN by microorganisms that can effectively promote the metabolism of nitrogen in microorganisms. Nitrogenase is a critical enzyme for converting atmospheric dinitrogen into ammonia. This enzyme is a 6-subunit composite enzyme system composed of molybdenum ferritin and ferritin, and its structure and function are highly conserved [170]. During the process of nitrogen fixation, electron carriers like ferredoxin first reduce iron, followed by the reduction of the catalytic component [57]. Nitrate can be reduced to nitrite by nitrate reductase (NR) and further to ammonia by NiR [171]. Finally, ammonia combines with glutamate under the action of GS and consumes ATP to generate glutamine, which generates α-ketoglutarate under the action of glutamate synthetase (GOGAT), which can be further converted to glutamate under the action of transaminase [87,172,173]. Glutamate is involved in protein synthesis as an amino acid residue in the presence of various enzymes and cofactors [174]. Although the mechanism of NPN assimilation is well understood, improving the NPN assimilation capacity of microorganisms remains a challenge. With the rapid advancements in systems biology and synthetic biology, researchers have begun utilizing genetic engineering tools to modify microorganisms to enhance their NPN assimilation capabilities. This progress largely involves the gene editing of key enzymes responsible for nitrogen assimilation and the optimization of metabolic pathways. Through genetic engineering techniques, scientists can boost the expression of nitrogen metabolism-related enzymes (such as nitrogenase) in microorganisms, thereby improving their ability to utilize nitrogen sources [175]. For example, the activity of nitrogenase increased tenfold by integrating nitrogenase genes from *A. vinelandii* into the *E. coli* chromosome and overexpressing genes associated with electron transfer and nitrogenase maturation [63]. Additionally, genes encoding heterologous related enzymes with good nitrogen-fixing capacity have been introduced into target species to reconfigure the nitrogen metabolism signaling pathway. For instance, 11 nitrogen assimilation genes from *Paenibacillus polymyxa* and two from *Klebsiella oxytoca* were successfully constructed and integrated into the rice genome using synthetic biology, leading to the stable expression and inheritance of the nitrogenase biosynthesis pathway in rice [176]. However, despite these advances in improving microbial NPN assimilation, several challenges persist. Firstly, the efficiency of microorganisms assimilating NPN is low under extreme conditions, such as high salinity and elevated temperatures, which limits their practical application. Secondly, while gene editing technology offers new methods for microorganism modification, achieving efficient, stable, and controllable gene modification remains a technical hurdle. Moreover, environmental factors (such as temperature, pH, and oxygen concentration) significantly influence the nitrogen assimilation efficiency of microorganisms, making it difficult to maintain a stable nitrogen assimilation process in complex industrial environments. Nonetheless, with ongoing breakthroughs in genomics, metabolic engineering, and synthetic biology technologies, there is hope for more efficient and sustainable microbial nitrogen assimilation applications, promoting broader applications in this field.

## 4. Industrial Fermentation Technology to Produce Feed Protein

**Figure 3:**
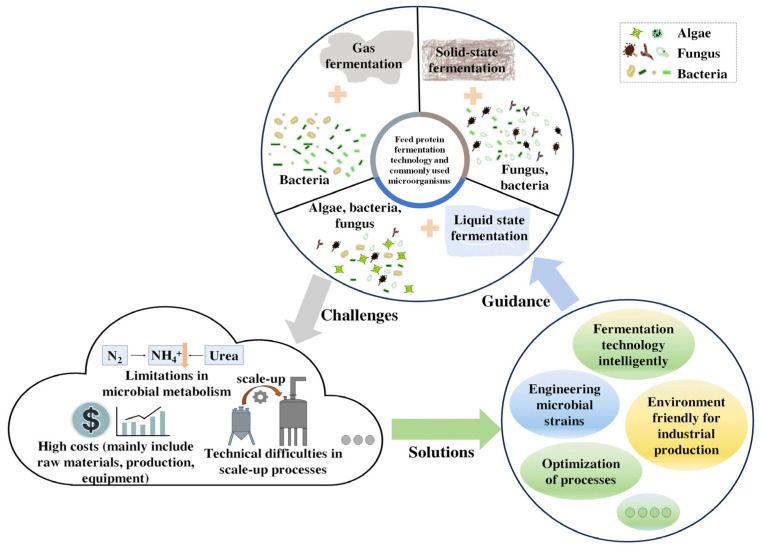
Industrial fermentation technologies and challenges for industrial production of feed protein: challenges and solutions.

Fermentation technology is at the heart of manufacturing industries, spanning pharmaceuticals, food, agriculture, biofuels, and environmental management [177]. The basic principle of industrial fermentation technology is to provide the necessary substrates and suitable conditions to satisfy microbial growth requirements. Generally, culture media containing carbon, nitrogen, salts, trace elements, and vitamins are essential for optimizing the fermentation process [31,178]. Microbial protein production through fermentation processes requires significant technological advances to deliver effective, stable, and safe products at scale, especially in the strict control of fermentation parameters, which may differ between microorganisms employed [18,179]. To convert NPN to feed protein effectively, it is critical to employ appropriate fermentation technology based on the strain and the form of NPN, which comprises solid state fermentation, liquid state fermentation, and gas fermentation (Figure 3).

### 4.1. Solid State Fermentation

Solid state fermentation (SSF) is a traditional technique that offers several advantages over liquid state fermentation, SSF: (a) the fermentation substrates are typically waste natural materials or agricultural by-products, which are widely available and cost-effective; (b) downstream processing is straightforward, and simply drying the fermented feeds is sufficient; and (c) anti-nutritional factors in the fermented feed can be effectively minimized, ensuring safety [180,181,182]. However, SSF faces challenges such as limited nutrient diffusion, difficulty in heat removal, and restricted microbial selection [14].

Substrates with high solid and insoluble content are commonly used in SSF for feed protein production, including agro-industrial wastes such as straw, corn husks, wheat bran, wine lees, cotton meal, soybean meal, and canola meal [166,183]. Generally, the feed protein of SSF is a mixture of fermented solid substrates and microbial cells. Rice straw pretreated with ammonia water and steam explosion treatment could be fermented by *A. niger* CICIMF 0410 and *Candida tropicalis* CICC 31949 in the solid state with a water content of 60% [184]. The crude and true protein contents of the rice straw increased by 4.37 and 5.03 times, respectively. The crude protein content of mango waste could reach 50.76% or 30.84% by *C. utilis* FMJ12 with a mixture of nutrient broth (10%, *w*/*v*) by adding 1% (*w*/*v*) of ammonium sulfate or ammonium nitrate and fermented in SSF [185]. The protein content of potato peels could be increased from 12.5% to 21.86% or 18.42% by *S. cerevisiae* with the supplement of 10 g N_2_/kg of ammonium sulfate or urea, respectively, in the SSF [186]. Our group recently focused on the SSF of agro-industrial processing wastes using artificially synthesized microbiomes to improve the protein content and nutritional value, which could fully deconstruct cellulose and convert NPN [187,188,189,190,191]. The microbial community, including *Agrobacterium rubi*, *Acinetobacter johnsonii*, *B. subtilis*, *Lactobacillus casei*, *Trichoderma viride*, *A. niger*, *Cladosporium cladosporioides*, *Sarocladium strictum*, could improve the absolute digestibility of true protein of corn stover supplemented with 4% (w/w) ammonium sulfate from 27.11% to 45.29% after 23 days of fermentation [189]. Distillers’ grains undergo aerobic-microaerophilic-anaerobic fermentation by *A. niger*, *S. cerevisiae*, *C. utilis*, *Rhodotorula benthica*, *Streptococcus thermophilus*, *Lactobacillus paracasei*, and *Lactobacillus fermentum*, the protein content and flavor were greatly improved. The true protein content improved from 10.81% to 16.44% under conditions of 50% moisture content, 1% urea addition (wet weight basis), and a fermentation time of 11 days [187]. Especially, the artificially evolved thermophilic microbial consortium could improve the true protein content of straw by 56–72% after only 7 days of SSF [188].

Bioreactors used for pilot and industrial-scale SSFs can be categorized into tray bioreactors, packed bed bioreactors, air pressure pulsation bioreactors, and intermittent or continuous hybrid bioreactors [192,193]. Tray and packed bed bioreactors are examples of static bioreactors suitable for the SSF of filamentous fungi, with no stirring and prevention of damage to the mycelium during fermentation [193]. Air pressure pulsation and intermittent or continuous hybrid bioreactors are classified as dynamic bioreactors. These systems feature various mechanical structures that facilitate the mixing of fermentation substrates while incorporating forced ventilation to enhance mass and heat transfer and promote the growth of microorganisms. The industrialization of SSF for feed proteins faces several challenges, including high costs, limitations in microbial metabolism, and difficulties in scaling up the process [194]. To address these issues, enhancing the robustness of the strains is a key strategy [195]. By optimizing relevant metabolic pathways, it is possible to increase the tolerance of strains to harsh environments, subsequently improving their metabolic efficiency. The development of more efficient reactors, such as drum fermentation systems and forced-vented reactors, could improve oxygen supply and temperature uniformity, thus enhancing the stability of industrial production [196]. Furthermore, accurately monitoring and controlling process parameters like pH, water content, oxygen levels, and product concentration is still a challenge for SSF. The emergence and development of intelligent and automated control technologies provide new insights for addressing these issues.

### 4.2. Liquid State Fermentation

Compared to SSF, liquid state fermentation (LSF) offers easier control over conditions such as temperature and pH, and the uniform distribution of nutrients in LSF supports large-scale production. However, the equipment required for LSF is expensive, consumes a lot of energy, and is susceptible to contamination from bacteria [197].

Generally, LSF uses soluble substrates to produce feed protein, in which the microbial cell is separated and collected, and the protein content is high (48%~71%) [14]. However, the cost of feed protein by LSF is higher than that of SSF because of the complex bioreactors and mixing systems [198]. Meanwhile, the abundant amino acids and multivitamins in microbial cells could provide greater nutritional value to animals. Many wastes in the liquid state include large amounts of carbohydrates or NPN, which have been explored for the production of microbial cells using LSF technology. Potato starch processing wastewater (PSPW), containing 488 mg/L of ammonium, was co-fermented by *C. utilis*, *Geotrichum candidum*, and *C. tropicalis*, resulting in a microbial protein yield of 3.06 g/L [199]. Soybean-processing wastewater can be fermented by a microbial community, including *Acidipropionibacterium* and *Propioniciclava*, and the collected microbial protein content can reach 47.8% [12]. In addition, some insoluble wastes can be treated to a soluble state for LSF. The large amounts of cellulose and hemicellulose in wheat straw can be hydrolyzed to soluble sugars by dilute acid pretreatment and enzymatic hydrolysis, which can be further fermented by *Trichosporon cutaneum* MP11 with supplementation of ammonium sulfate (24 g/L). The protein yield of the LSF of hydrolyzed wheat straw can reach 24.4 g/L after 48 h [200]. The hydrolyzed pineapple peel added with 2 g/L of ammonium oxalate could be fermented by *T. viride* ATCC28038, and the protein content was increased from 9.44 mg/mL to 55.44 mg/mL [201].

Equipment of LSF includes airlift bioreactors, stirred-tank bioreactors, and bubble column bioreactors [202]. Stirred-tank bioreactors are known for their high oxygen transfer efficiency and are suitable for the high-density culture of microorganisms, although they tend to consume significant amounts of energy. In contrast, airlift bioreactors consume less energy but have limited oxygen transfer efficiency. Bubble column bioreactors have a simpler structure compared to airlift bioreactors while exhibiting lower oxygen transfer efficiency. Among these, stirred-tank bioreactors are commonly used in the production of mycoprotein [203]. The industrial production of LSF faces challenges such as uneven nutrient distribution during large-scale processes and high production costs. Optimizing bioreactor equipment and implementing circular economy models offer promising solutions to these problems. Controlling the various fermentation parameters (e.g., temperature, pH, and dissolved oxygen) that strongly influence microbial growth and metabolism is a persistent challenge in large-scale liquid fermentation. Therefore, enhancing the stability and adaptability of microorganisms for sustained large-scale production is a long-term objective. Meanwhile, the risk of microbial contamination remains a significant issue in LSF [204]. With advances in bioengineering and automation control technology, the industrialized production of LSF is expected to overcome these challenges in the future.

### 4.3. Gas Fermentation

A variety of large-scale industrial processes, such as those in refineries, steel mills, and ferroalloy industries, generate industrial waste gases that can drastically increase the greenhouse gas load in the atmosphere. Microorganisms can utilize these exhaust gases through gaseous fermentation (GF) [205]. However, only certain microorganisms can utilize these gases, including hydrogen-oxidizing bacteria, methane-oxidizing bacteria, and carbon monoxide-oxidizing bacteria [206].

The production process of feed protein from gas includes gas collection and pretreatment, microbial fermentation, product recovery, and processing. The production of microbial cells is always accompanied by the synthesis of other biochemical bioproducts, such as ethanol, biodiesel, and glycerol [207]. The strains *Moorella thermoacetica* and *C. necator* can metabolize steel mill exhaust CO and NH_3_, and the cost of microbial protein is only 2.78 USD/kg, which is much lower than the benchmark model of a unit production cost of 4.15 USD/kg of protein [208]. Calysta Corporation produced a microbial protein with a protein content exceeding 70% by methane-oxidizing bacteria using a mixture of methane, oxygen, and atmospheric dinitrogen as raw materials. The feed protein of Calysta Corporation has been applied to salmon farming and has already been marketed in the European Union. Beijing Shoulang Biotechnology Co., Ltd. (Beijing, China) produced a microbial protein with a crude protein content of 80% to 92.4% using *Clostridium autoethanogenum* and CO, CO_2_, and ammonia water as fermentation substrates. This protein has been used as a substitute for fishmeal in aquaculture [209]. In addition, companies such as Solarfoods, Kiverdi, Inc., Novo Nutrients, and Avecom. produce microbial proteins by fermenting a mixture of CO_2_ and H_2_ with hydrogen-oxidizing bacteria, which are widely used in aquaculture [209].

The equipment used for GF includes stirred tank, microbubble, Taylor-Couette vortex, torus, airlift, membrane bioreactors, moving bed biofilm, trickling bed reactors, and U-loop fermenters [210]. Stirred-tank reactors, airlift reactors, and U-loop fermenters are suitable for large-scale industrial applications because of their high gas transfer efficiency. Different types of fermenters or bioreactors are required depending on the type of microorganism and fermentation method. In practice, the conversion of industrial gas into feed protein requires efficient and stable technology. Meanwhile, these products may not necessarily have economic advantages over conventional feed proteins, which is a major obstacle to their widespread adoption and application. The design of fermenters, manipulation of gases, and nitrogen fixation capability of microorganisms are the main challenges for GF. It is essential to have specialized reactor system designs, particularly for equipment that operates under high-pressure and high-temperature gases [211]. This GF equipment must ensure high gas solubility and be equipped for effective gas transfer. Constructing a sensitive sensor system within the microorganism can improve its capacity to sense nitrogen during the GF [212]. Additionally, optimizing the nitrogen cycling pathway and establishing an efficient “nitrogen capture-nitrogen fixation-nitrogen cycling” model can further boost feed protein production.

## 5. Evolution and Screening of Industrial Strains Enhancing the Assimilation of Non-Protein Nitrogen

Efficient assimilation of NPN is essential for the production of feed proteins by microorganisms. The strains added directly into feed should be used strictly by regulations, which are normally limited to dozens of certain species [213]. For example, 35 strains can be directly fed or used in feed fermentation based on the “Regulations on Feed and Feed Additives” in China. Although some native microorganisms can utilize NPN, naturally isolated microorganisms can rarely be directly used for industrial-scale production because of their low yields and weak tolerance to harsh industrial conditions [214]. To increase the protein yield and robustness of the strain, several rational engineering, random mutagenesis, and adaptive laboratory evolution (ALE) approaches have been developed [215]. However, rational engineering of most native strains is difficult and time-consuming due to the lack of advanced genetic tools and metabolic knowledge [216,217]. Moreover, the direct use of engineering strains in feed faces challenges, as governments around the world have formulated strict laws and regulations to supervise the application of engineering strains in feed, and some people cannot accept artificial genetic engineering technology in feed or food [218,219].

Random mutagenesis typically involves exposing strains to physical (such as ultraviolet, α-rays, β-rays, γ-rays, and X-rays) or chemical (alkylating agents, alkali analogs, and antibiotics) mutagens multiple times to create genetic and phenotypic diversity [220]. Recently, atmospheric pressure room temperature plasma (ARTP) and heavy particle line irradiation have been attracting a great deal of attention because of their high mutation rate, mutation diversity, and ease of operation [221,222]. The protein content of the mutant *Auxenochlorella pyrenoidosa* MMC-8 obtained by ARTP was increased to 63.26% from 40.11%, and the protein productivity reached 0.87 g/L/d [223]. The mycelial growth rate of mutant *Pleurotus djamor* 240S-4 obtained by ARTP mutagenesis increased from 4 mm/day to 9.5 mm/day, and the protein content increased by 28% [224]. Research on the enhancement of microbial protein production through heavy particle line irradiation is limited, although this method has been applied to boost the production of other metabolites. Yongjuan Liu et al. treated *Coleophoma empetri* MEFC09 with heavy-ion irradiation, which significantly increased the production of the micafungin precursor FR901379 from 0.2–0.3 g/L to 1.1 g/L [225]. However, the probability of a beneficial mutation can be very low (<1/10^5^), and the efficiency of conventional screening is low throughput, leading to high costs for screening large numbers of mutants [221]. Thus, it is crucial to develop high-throughput screening (HTS) methods for rapidly screening microbial strains in a large library of mutants, which combine automated and micro-quantitative experiments with the analysis of large-scale data [226]. Automated steps include sampling, dilution of samples to a suitable range for detection, mixing samples, washing cells, development of chromogenic or fluorescence detection, data analysis, and collection of the targeted strains. In the recent decade, HTS has been combined with droplet-based microfluidics, which can be called ultra-HTS. The (ultra-) HTS requires few human resources and only several microliters (in microplates) or even nanoliters (in droplets), leading to a significant decrease in costs.

ALE is a natural evolution of microorganisms under controlled laboratory conditions, which is driven by beneficial mutations [227,228,229]. In contrast to natural evolution, ALE evolves in a set direction with artificial control [227]. Compared to genetic modification engineering, ALE has the advantage of regulating many different genes in parallel without introducing other genes [230]. Especially, ALE can be used to improve the specific characteristics of microbial communities [231]. The microbial community can mutually utilize the metabolites produced by each other and thus has stronger adaptability to waste, including complex components than single strains [232]. Numerous studies have reported the use of ALE to optimize microbial metabolic pathways, restore growth rates, increase tolerance to unfavorable factors, and increase the yield of target products [233,234]. The growth rate of *E. coli* K-12 MG1655 on glucose minimal media increased from 0.69 h^−1^ to 1.01 h^−1^ through ALE [235]. The doubling time of *E. coli* FMX892 obtained by ALE was shortened from 79.2 to 4.5 h, which was close to the natural *E. coli* growth rate [236]. The application of ALE to improve NPN assimilation ability or protein yield has not been reported in the literature.

## 6. Conclusions and Further Perspective

Microbial protein produced from NPN is a promising alternative to plant-sourced protein (e.g., soybean) for animal feed, which avoids competition for food between people and animals. To date, many researchers have conducted feed protein production from different microbial sources, including microalgae, yeast, fungi, and bacteria, which use various industrial and agricultural wastes as substrates. Meanwhile, many reviews have focused on deconstructing cellulose and hemicellulose in waste to provide enough accessible carbon sources for microbes. This review provides a useful yet concise update on industrial microbial technologies in the application of NPN for feed protein production. To improve the cost and efficiency of microbial production, it is critical to provide microorganisms with low-cost nitrogen sources (e.g., atmospheric dinitrogen, urea, and ammonia) or waste containing sufficient nitrogen. With advances in the synthesis mechanism of proteins in microbial cells, NPN assimilation efficiency can be improved either by fermentation technology or novel strain creation. For feed protein production on a large scale, the fermentation process has been largely explored from the viewpoint of equipment and technology. Additionally, strain is the core of the fermentation process and the main contributor of the protein, which could be evolved by random mutagenesis and ALE approaches and then screened by the recently developed (ultra-) high-throughput screening method.

From the viewpoint of technology, the above advancements in various areas could promise to remove relevant obstacles for efficient microbial protein production on a large scale. However, the wide commercial production of microbial protein for animal feed can only be made possible by synergistic efforts regarding safety, regulation, and popular acceptance. Currently, consumer awareness of feed proteins is low, especially due to the lack of understanding of their positive impacts on animal health, growth, and development. In addition, the commercial production of microbial proteins still faces many challenges. Firstly, further investigation is required to explore the implementation of various NPNs and strains for microbial protein production. Meanwhile, it is necessary to manage the synchronous assessment of microbial protein used for feed by evaluating its potential risks and safety. Secondly, with the rapid development and wide application of synthetic biology technology, the genetic modification of microorganisms has become an effective means of enhancing the nitrogen utilization capacity. However, with the strict restrictions of current policies and regulations, the compliance issue of transgenic manipulation of microorganisms has become an important challenge in industrial feed protein production. Improving the policy environment and strengthening compliance management are important ways to comply with regulations for compliant production. Economic efficiency can be enhanced by optimizing raw material selection and energy usage. For example, the reuse of by-products or wastewater can not only reduce energy consumption and environmental pollution but also create additional economic benefits. Based on various optimization strategies, cost reduction can be effectively achieved, and large-scale production can be realized. In any case, technical issues, quality assurance, safety, regulatory, and environmental impact have to be duly addressed to expand the microbial protein in the feed protein market, which will alleviate the shortage of plant-sourced feed protein and avoid the loss and environmental risk of NPN.

## Figures and Tables

**Table 1:** Wastes containing non-protein nitrogen (NPN).

NPN Type	Waste Sources	Concentration	Reference
Ammonia and ammonium salts	Food waste (average)	0.76 g/kg	[132]
	Anaerobic fermentation digestate food waste	1.1~9.6 kg N/t	[133]
	Hydrolysates of food waste	1081 mg/L	[134]
	Digestate of Wastewater treatment plant	4040.74 mg/L	[135]
	Municipal wastewater	100 mg/L	[136]
	Anaerobic digestion effluents of municipal sewage sludge	1540 mg/L	[137]
	Wastewater treatment plant treated water	35 mg/L	[138]
	Anaerobically digested sludge from wastewater treatment plant	318.80 mg/L	[139]
	Liquid digestate from swine farm	532 mg/L	[140]
	Chicken manure	2937 mg/L	[141]
Urea	Cattle urine	50~438.3 g/kg	[142]

## Data Availability

No new data were created or analyzed in this study.

## Abbreviations

The following abbreviations are used in this manuscript:

NPN	Non-protein nitrogen
RDA	Recommended Dietary Allowance
UCYN-A	*Candidatus* Atelocyanobacter thalassa
KBM	KnipBio Meal
FDA	Food and Drug Administration
MAFF	Ministry of Agriculture, Forestry, and Fisheries
GFMP	Good Feed Manufacturing Practice
NR	Nitrate reductase
NiR	Nitrite reductase
GS	Glutamine synthetase
GOGAT	Glutamate synthetase
UC	Urea carboxylase
Cu-NiRs	Copper-type nitrite reductases
cd1NiRs	Cytochrome cd1-type nitrite reductases
ccNiRs	Multiheme nitrite reductase
SSF	Solid state fermentation
LSF	Liquid state fermentation
GF	Gas fermentation
ALE	Adaptive laboratory evolution
ARTP	Atmospheric pressure room temperature plasma
HTS	High-throughput screening

## References

[1] Lonnie M., Hooker E., Brunstrom J., Corfe B., Green M., Watson A., Williams E., Stevenson E., Penson S., Johnstone A. (2018). Protein for Life: Review of Optimal Protein Intake, Sustainable Dietary Sources and the Effect on Appetite in Ageing Adults. Nutrients.

[2] Smith K., Watson A.W., Lonnie M., Peeters W.M., Oonincx D., Tsoutsoura N., Simon-Miquel G., Szepe K., Cochetel N., Pearson A.G. (2024). Meeting the Global Protein Supply Requirements of a Growing and Ageing Population. Eur. J. Nutr..

[3] Ferrari L., Panaite S.-A., Bertazzo A., Visioli F. (2022). Animal- and Plant-Based Protein Sources: A Scoping Review of Human Health Outcomes and Environmental Impact. Nutrients.

[4] Liu Y., Aimutis W.R., Drake M. (2024). Dairy, Plant, and Novel Proteins: Scientific and Technological Aspects. Foods.

[5] Smil V. (2002). Nitrogen and Food Production: Proteins for Human Diets. AMBIO A J. Hum. Environ..

[6] Clark M., Tilman D. (2017). Comparative Analysis of Environmental Impacts of Agricultural Production Systems, Agricultural Input Efficiency, and Food Choice. Environ. Res. Lett..

[7] Sodiq A., Baloch A.A.B., Khan S.A., Sezer N., Mahmoud S., Jama M., Abdelaal A. (2019). Towards Modern Sustainable Cities: Review of Sustainability Principles and Trends. J. Clean. Prod..

[8] Boland M.J., Rae A.N., Vereijken J.M., Meuwissen M.P.M., Fischer A.R.H., Van Boekel M.A.J.S., Rutherfurd S.M., Gruppen H., Moughan P.J., Hendriks W.H. (2013). The Future Supply of Animal-Derived Protein for Human Consumption. Trends Food Sci. Technol..

[9] Makkar H.P.S. (2018). Review: Feed Demand Landscape and Implications of Food-Not Feed Strategy for Food Security and Climate Change. Animal.

[10] Kim S.W., Less J.F., Wang L., Yan T., Kiron V., Kaushik S.J., Lei X.G. (2019). Meeting Global Feed Protein Demand: Challenge, Opportunity, and Strategy. Annu. Rev. Anim. Biosci..

[11] Parisi G., Tulli F., Fortina R., Marino R., Bani P., Dalle Zotte A., De Angelis A., Piccolo G., Pinotti L., Schiavone A. (2020). Protein Hunger of the Feed Sector: The Alternatives Offered by the Plant World. Ital. J. Anim. Sci..

[12] Santillan E., Yasumaru F., Vethathirri R.S., Thi S.S., Hoon H.Y., Sian D.C.P., Wuertz S. (2024). Microbial Community-Based Protein from Soybean-Processing Wastewater as a Sustainable Alternative Fish Feed Ingredient. Sci. Rep..

[13] Xu X., Sharma P., Shu S., Lin T.-S., Ciais P., Tubiello F.N., Smith P., Campbell N., Jain A.K. (2021). Global Greenhouse Gas Emissions from Animal-based Foods are Twice those of Plant-based Foods. Nat. Food.

[14] Li Y.P., Ahmadi F., Kariman K., Lackner M. (2024). Recent Advances and Challenges in Single Cell Protein (SCP) Technologies for Food and Feed Production. npj Sci. Food.

[15] Ayodele T., Tijani A., Liadi M., Alarape K., Clementson C., Hammed A. (2024). Biomass-Based Microbial Protein Production: A Review of Processing and Properties. Front. Biosci.-Elite.

[16] Nadar C.G., Fletcher A., Moreira B.R.D.A., Hine D., Yadav S. (2024). Waste to Protein: A Systematic Review of a Century of Advancement in Microbial Fermentation of Agro-industrial Byproducts. Compr. Rev. Food Sci. Food Saf..

[17] Bojana B., Vucurovic D., Vasic D., Jevtic M.R., Dodic S. (2022). Biotechnological Production of Sustainable Microbial Proteins from Agro-Industrial Residues and By-Products. Foods.

[18] Muniz E.D.N., Montenegro R.T.D.Q., Da Silva D.N., D’Almeida A.P., Gonçalves L.R.B., De Albuquerque T.L. (2024). Advances in Biotechnological Strategies for Sustainable Production of Non-Animal Proteins: Challenges, Innovations, and Applications. Fermentation.

[19] Chama N.T. (2019). Production of Single-Cell Protein from Different Substrates. Aust. J. Sci. Technol..

[20] Xie R., Wang Y., Chen Q., Guo W., Jiao N., Zheng Q. (2020). Coupling Between Carbon and Nitrogen Metabolic Processes Mediated by Coastal Microbes in *Synechococcus*-Derived Organic Matter Addition Incubations. Front. Microbiol..

[21] Bibra M., Samanta D., Sharma N.K., Singh G., Johnson G.R., Sani R.K. (2022). Food Waste to Bioethanol: Opportunities and Challenges. Fermentation.

[22] Jagannathan P., Muthukumaran C., Tamilarasan K. (2017). A Sequential Pretreatment of Lignocelluloses in Bamboo Biomass to Fermentable Sugars by Acid/Enzymatic Hydrolysis. 3 Biotech.

[23] Alawad I., Ibrahim H. (2024). Pretreatment of Agricultural Lignocellulosic Biomass for Fermentable Sugar: Opportunities, Challenges, and Future Trends. Biomass Convers. Biorefinery.

[24] Begum W., Saha B., Mandal U. (2023). A Comprehensive Review on Production of Bio-Surfactants by Bio-Degradation of Waste Carbohydrate Feedstocks: An Approach towards Sustainable Development. RSC Adv..

[25] Ahamefule C.S., Osilo C., Ahamefule B.C., Madueke S.N., Moneke A.N. (2024). Simultaneous Production of Biofuel from Agricultural Wastes and Bioremediation of the Waste Substrates: A Review. Curr. Res. Microb. Sci..

[26] Gao L., Liu X. (2010). Effects of Carbon Concentrations and Carbon to Nitrogen Ratios on Sporulation of Two Biological Control Fungi as Determined by Different Culture Methods. Mycopathologia.

[27] Manyi-Loh C.E., Lues R. (2023). Anaerobic Digestion of Lignocellulosic Biomass: Substrate Characteristics (Challenge) and Innovation. Fermentation.

[28] Hadiyarto A., Soetrisnanto D., Rosyidin I., Fitriana A. (2019). Co-Digestion of Bagasse and Waterhyacinth for Biogas Production with Variation of C/N and Activated Sludge. J. Phys. Conf. Ser..

[29] Panda J., Amrit R., Mishra A.K., Chakraborty A., Rustagi S., Nath P.C., Sarabandi K., Sarma H., Wagh M.S., Mohanta Y.K. (2025). Sustainable Valorization of Fruit and Vegetable Waste for Bioactive Compounds: Advancing Functional Food and Wellness. Waste Biomass Valor.

[30] Guida S., Van Peteghem L., Luqmani B., Sakarika M., McLeod A., McAdam E.J., Jefferson B., Rabaey K., Soares A. (2022). Ammonia Recovery from Brines Originating from a Municipal Wastewater Ion Exchange Process and Valorization of Recovered Nitrogen into Microbial Protein. Chem. Eng. J..

[31] Olsen P.M., Horn S.J., Byrtusova D., Moen L.F., Shapaval V., Hansen L.D. (2025). Assessment of Different Nitrogen Sources and Bioreactor Cultivation Strategies during Growth of *Aurantiochytrium Limacinum* on Spruce Sugars. Algal Res..

[32] Dewhurst R.J., Newbold J.R. (2022). Effect of Ammonia Concentration on Rumen Microbial Protein Production In Vitro. Britisb J. Nutr..

[33] Ijaola A.O., Akamo D.O., George T.T., Sengul A., Adediji M.Y., Asmatulu E. (2024). Algae as a Potential Source of Protein: A Review on Cultivation, Harvesting, Extraction, and Applications. Algal Res..

[34] Nandy S.K., Srivastava R.K. (2018). A Review on Sustainable Yeast Biotechnological Processes and Applications. Microbiol. Res..

[35] Koch H., Sessitsch A. (2024). The Microbial-Driven Nitrogen Cycle and Its Relevance for Plant Nutrition. J. Exp. Bot..

[36] Li J., Yuan M., Meng N., Li H., Sun J., Sun B. (2024). Influence of Nitrogen Status on Fermentation Performances of Non- *Saccharomyces* Yeasts: A Review. Food Sci. Hum. Wellness.

[37] Chen Y., Lin Y., Zhu J., Zhou J., Lin H., Fu Y., Zhou Y. (2024). Transcriptomic Analysis of Nitrogen Metabolism Pathways in *Klebsiella Aerogenes* Under Nitrogen-Rich Conditions. Front. Microbiol..

[38] Baumann K.B.L., Mazzoli A., Salazar G., Ruscheweyh H.-J., Müller B., Niederdorfer R., Sunagawa S., Lever M.A., Lehmann M.F., Bürgmann H. (2024). Metagenomic and -Transcriptomic Analyses of Microbial Nitrogen Transformation Potential, and Gene Expression in Swiss Lake Sediments. ISME Commun..

[39] Rojo M.C., Talia P.M., Lerena M.C., Ponsone M.L., Gonzalez M.L., Becerra L.M., Mercado L.A., Martín-Arranz V., Rodríguez-Gómez F., Arroyo-López F.N. (2023). Evaluation of Different Nitrogen Sources on Growth and Fermentation Performance for Enhancing Ethanol Production by Wine Yeasts. Heliyon.

[40] Tang S., Pan W., Zhou J., Ma Q., Yang X., Wanek W., Marsden K.A., Kuzyakov Y., Chadwick D.R., Wu L. (2024). Soil Nitrogen and Phosphorus Regulate Decomposition of Organic Nitrogen Compounds in the Rothamsted Experiment. Soil Biol. Biochem..

[41] Hu C.-C., Liu X.-Y., Driscoll A.W., Kuang Y.-W., Brookshire E.N.J., Lü X.-T., Chen C.-J., Song W., Mao R., Liu C.-Q. (2024). Global Distribution and Drivers of Relative Contributions among Soil Nitrogen Sources to Terrestrial Plants. Nat. Commun..

[42] Jin D., Zhao S., Zheng N., Beckers Y., Wang J. (2018). Urea Metabolism and Regulation by Rumen Bacterial Urease in Ruminants–A Review. Ann. Anim. Sci..

[43] Dixon R., Kahn D. (2004). Genetic Regulation of Biological Nitrogen Fixation. Nat. Rev. Microbiol..

[44] Massana R. (2024). The Nitroplast: A Nitrogen-Fixing Organelle. Science.

[45] Guo K., Yang J., Yu N., Luo L., Wang E. (2023). Biological Nitrogen Fixation in Cereal Crops: Progress, Strategies, and Perspectives. Plant Commun..

[46] Dai H., Wei S., Li J., Kong W., Wang B., Pei J., Wu J. (2024). Fertilization Effects on Symbiotic and Free-Living Biological Nitrogen Fixations: Similar Effects but Different Mechanisms. Appl. Soil Ecol..

[47] Aasfar A., Bargaz A., Yaakoubi K., Hilali A., Bennis I., Zeroual Y., Meftah Kadmiri I. (2021). Nitrogen Fixing *Azotobacter* Species as Potential Soil Biological Enhancers for Crop Nutrition and Yield Stability. Front. Microbiol..

[48] Wang H., Zhang L., Tian C., Fan S., Zheng D., Song Y., Gao P., Li D. (2024). Effects of Nitrogen Supply on Hydrogen-Oxidizing Bacterial Enrichment to Produce Microbial Protein: Comparing Nitrogen Fixation and Ammonium Assimilation. Bioresour. Technol..

[49] Hu X., Vandamme P., Boon N. (2022). Co-Cultivation Enhanced Microbial Protein Production Based on Autotrophic Nitrogen-Fixing Hydrogen-Oxidizing Bacteria. Chem. Eng. J..

[50] Hu X., Kerckhof F.-M., Ghesquière J., Bernaerts K., Boeckx P., Clauwaert P., Boon N. (2020). Microbial Protein out of Thin Air: Fixation of Nitrogen Gas by an Autotrophic Hydrogen-Oxidizing Bacterial Enrichment. Environ. Sci. Technol..

[51] Masson-Boivin C. (2018). Symbiotic Nitrogen Fixation by Rhizobia—The Roots of a Success Story. Curr. Opin. Plant Biol..

[52] Zhang W., Chen Y., Huang K., Wang F., Mei Z. (2023). Molecular Mechanism and Agricultural Application of the NifA–NifL System for Nitrogen Fixation. Int. J. Mol. Sci..

[53] Mus F., Crook M.B., Garcia K., Garcia Costas A., Geddes B.A., Kouri E.D., Paramasivan P., Ryu M.-H., Oldroyd G.E.D., Poole P.S. (2016). Symbiotic Nitrogen Fixation and the Challenges to Its Extension to Nonlegumes. Appl. Environ. Microbiol..

[54] Westhead O., Barrio J., Bagger A., Murray J.W., Rossmeisl J., Titirici M.-M., Jervis R., Fantuzzi A., Ashley A., Stephens I.E.L. (2023). Near Ambient N2 Fixation on Solid Electrodes versus Enzymes and Homogeneous Catalysts. Nat. Rev. Chem..

[55] Riyaz Z., Khan S.T. (2025). Nitrogen Fixation by Methanogenic Archaea, Literature Review and DNA Database-Based Analysis; Significance in Face of Climate Change. Arch. Microbiol..

[56] Kartal B., Keltjens J.T. (2016). Anammox Biochemistry: A Tale of Heme c Proteins. Trends Biochem. Sci..

[57] Ettwig K.F., Butler M.K., Le Paslier D., Pelletier E., Mangenot S., Kuypers M.M.M., Schreiber F., Dutilh B.E., Zedelius J., De Beer D. (2010). Nitrite-Driven Anaerobic Methane Oxidation by Oxygenic Bacteria. Nature.

[58] Fang F.C. (2004). Antimicrobial Reactive Oxygen and Nitrogen Species: Concepts and Controversies. Nat. Rev. Microbiol..

[59] Bennett E.M., Murray J.W., Isalan M. (2023). Engineering Nitrogenases for Synthetic Nitrogen Fixation: From Pathway Engineering to Directed Evolution. BioDesign Res..

[60] Han Y., Lv M., Liu J., He S., Shi W., Li M., Gao Z. (2025). Agronomic Practices-Driven Response of Nitrogen-Related Microorganisms. Plant Soil.

[61] Michel-Reydellet N., Kaminski P.A. (1999). *Azorhizobium caulinodans* P_II_ and GlnK Proteins Control Nitrogen Fixation and Ammonia Assimilation. J. Bacteriol..

[62] Schnabel T., Sattely E. (2021). Engineering Posttranslational Regulation of Glutamine Synthetase for Controllable Ammonia Production in the Plant Symbiont *Azospirillum brasilense*. Appl. Environ. Microbiol..

[63] Ito Y., Yoshidome D., Hidaka M., Araki Y., Ito K., Kosono S., Nishiyama M. (2024). Improvement of the Nitrogenase Activity in *Escherichia coli* That Expresses the Nitrogen Fixation-Related Genes from *Azotobacter vinelandii*. Biochem. Biophys. Res. Commun..

[64] Tatemichi Y., Nakahara T., Ueda M., Kuroda K. (2021). Construction of Recombinant *Escherichia coli* Producing Nitrogenase-Related Proteins from *Azotobacter vinelandii*. Biosci. Biotechnol. Biochem..

[65] Yang Z., Han Y., Ma Y., Chen Q., Zhan Y., Lu W., Cai L., Hou M., Chen S., Yan Y. (2018). Global Investigation of an Engineered Nitrogen-Fixing *Escherichia coli* Strain Reveals Regulatory Coupling between Host and Heterologous Nitrogen-Fixation Genes. Sci. Rep..

[66] Brown K.A., Harris D.F., Wilker M.B., Rasmussen A., Khadka N., Hamby H., Keable S., Dukovic G., Peters J.W., Seefeldt L.C. (2016). Light-Driven Dinitrogen Reduction Catalyzed by a CdS:Nitrogenase MoFe Protein Biohybrid. Science.

[67] Dey S., Awata T., Mitsushita J., Zhang D., Kasai T., Matsuura N., Katayama A. (2021). Promotion of Biological Nitrogen Fixation Activity of an Anaerobic Consortium Using Humin as an Extracellular Electron Mediator. Sci. Rep..

[68] Luo Y.W., Lou Y.W., Shi D., Kranz S.A., Hopkinson B.M., Hong H., Shen R., Zhang F. (2019). Reduced Nitrogenase Efficiency Dominates Response of the Globally Important Nitrogen Fixer *Trichodesmium* to Ocean Acidification. Nat. Commun..

[69] Azeem B., KuShaari K., Man Z.B., Basit A., Thanh T.H. (2014). Review on Materials & Methods to Produce Controlled Release Coated Urea Fertilizer. J. Control. Release.

[70] Foschi F.G. (2015). Urea Cycle Disorders: A Case Report of a Successful Treatment with Liver Transplant and a Literature Review. World J. Gastroenterol..

[71] Mobley H.L., Hausinger R.P. (1989). Microbial Ureases: Significance, Regulation, and Molecular Characterization. Microbiol. Rev..

[72] Patra A.K., Aschenbach J.R. (2018). Ureases in the Gastrointestinal Tracts of Ruminant and Monogastric Animals and Their Implication in Urea-N/Ammonia Metabolism: A Review. J. Adv. Res..

[73] González-Martín I., Hernández-Hierro J.M. (2008). Detection and Quantification of Additives (Urea, Biuret and Poultry Litter) in Alfalfas by Nir Spectroscopy with Fibre-Optic Probe. Talanta.

[74] Inácio A.G., Ítavo C.C.B.F., Dias A.M., Dos Santos Difante G., De Queiroz J.F., De Oliveira L.C.S., Dos Santos G.T., Ítavo L.C.V. (2022). A New Feed Additive Composed of Urea and Soluble Carbohydrate Coated with Wax for Controlled Release in Ruminal Fluid. Sci. Rep..

[75] Esteves E.A., Martino H.S.D., Oliveira F.C.E., Bressan J., Costa N.M.B. (2010). Chemical Composition of a Soybean Cultivar Lacking Lipoxygenases (LOX2 and LOX3). Food Chem..

[76] Suriyapha C., Suntara C., Wanapat M., Cherdthong A. (2022). Effects of Substituting Agro-Industrial by-Products for Soybean Meal on Beef Cattle Feed Utilization and Rumen Fermentation. Sci. Rep..

[77] Tang Z., Zhang J., Yuan X., Wang D., Luo H., Yang R., Wang H. (2025). Urea Promotes Alkaline Anaerobic Fermentation of Waste Activated Sludge for Hydrogen Production. Bioresour. Technol..

[78] Tourna M., Stieglmeier M., Spang A., Könneke M., Schintlmeister A., Urich T., Engel M., Schloter M., Wagner M., Richter A. (2011). *Nitrososphaera viennensis*, an Ammonia Oxidizing Archaeon from Soil. Proc. Natl. Acad. Sci. USA.

[79] Strope P.K., Nickerson K.W., Harris S.D., Moriyama E.N. (2011). Molecular Evolution of Urea Amidolyase and Urea Carboxylase in Fungi. BMC Evol. Biol..

[80] Konzock O., Zaghen S., Fu J., Kerkhoven E.J. (2022). Urea Is a Drop-in Nitrogen Source Alternative to Ammonium Sulphate in *Yarrowia lipolytica*. iScience.

[81] Brabender M., Hussain M.S., Rodriguez G., Blenner M.A. (2018). Urea and Urine Are a Viable and Cost-Effective Nitrogen Source for *Yarrowia lipolytica* Biomass and Lipid Accumulation. Appl. Microbiol. Biotechnol..

[82] Li Z., Wang D., Shi Y.-C. (2017). Effects of Nitrogen Source on Ethanol Production in Very High Gravity Fermentation of Corn Starch. J. Taiwan Inst. Chem. Eng..

[83] Chan-u-tit P., Laopaiboon L., Jaisil P., Laopaiboon P. (2013). High Level Ethanol Production by Nitrogen and Osmoprotectant Supplementation Under Very High Gravity Fermentation Conditions. Energies.

[84] Afrasiab K.T., Noppawan D., Imrana N.S., Nicom L., Sarote S., Wirat V., Pramuk P. (2021). Utilization of Urea as a Nitrogen Source for Ethanol Production from Oil Palm Trunk Using Simultaneous Saccharification and Fermentation. Agric. Nat. Resour..

[85] Zhao G., Zhang W., Zhang G. (2010). Production of Single Cell Protein Using Waste Capsicum Powder Produced during Capsanthin Extraction. Lett. Appl. Microbiol..

[86] Kumar A., Bera S. (2020). Revisiting Nitrogen Utilization in Algae: A Review on the Process of Regulation and Assimilation. Bioresour. Technol. Rep..

[87] Su Y. (2021). Revisiting Carbon, Nitrogen, and Phosphorus Metabolisms in Microalgae for Wastewater Treatment. Sci. Total Environ..

[88] Rosa R.M., Machado M., Vaz M.G.M.V., Lopes-Santos R., Nascimento A.G.D., Araújo W.L., Nunes-Nesi A. (2023). Urea as a Source of Nitrogen and Carbon Leads to Increased Photosynthesis Rates in *Chlamydomonas reinhardtii* Under Mixotrophy. J. Biotechnol..

[89] Ramanna L., Guldhe A., Rawat I., Bux F. (2014). The Optimization of Biomass and Lipid Yields of *Chlorella sorokiniana* When Using Wastewater Supplemented with Different Nitrogen Sources. Bioresour. Technol..

[90] Podevin M., De Francisci D., Holdt S.L., Angelidaki I. (2015). Effect of Nitrogen Source and Acclimatization on Specific Growth Rates of Microalgae Determined by a High-Throughput in Vivo Microplate Autofluorescence Method. J. Appl. Phycol..

[91] Veaudor T., Cassier-Chauvat C., Chauvat F. (2019). Genomics of Urea Transport and Catabolism in Cyanobacteria: Biotechnological Implications. Front. Microbiology..

[92] Hausinger R.P. (2004). Metabolic Versatility of Prokaryotes for Urea Decomposition. J. Bacteriol..

[93] Hailemariam S., Zhao S., He Y., Wang J. (2021). Urea Transport and Hydrolysis in the Rumen: A Review. Anim. Nutr..

[94] He H., Li Y., Zhang L., Ding Z., Shi G. (2023). Understanding and Application of *Bacillus* Nitrogen Regulation: A Synthetic Biology Perspective. J. Adv. Res..

[95] Shi Y., Niu X., Yang H., Chu M., Wang N., Bao H., Zhan F., Yang R., Lou K. (2024). Optimization of the Fermentation Media and Growth Conditions of *Bacillus velezensis* BHZ-29 Using a Plackett–Burman Design Experiment Combined with Response Surface Methodology. Front. Microbiol..

[96] Yang P., Chen Y., Gong A. (2021). Development of a Defined Medium for *Corynebacterium glutamicum* Using Urea as Nitrogen Source. 3 Biotech.

[97] Li J., Zhang J., Huang W., Kong F., Li Y., Xi M., Zheng Z. (2016). Comparative Bioavailability of Ammonium, Nitrate, Nitrite and Urea to Typically Harmful Cyanobacterium *Microcystis aeruginosa*. Mar. Pollut. Bull..

[98] Sun R., Li W., Hu C., Liu B. (2019). Long-Term Urea Fertilization Alters the Composition and Increases the Abundance of Soil Ureolytic Bacterial Communities in an Upland Soil. FEMS Microbiol. Ecol..

[99] Zhu J., Chen W., Chen H., Zhang X., He C., Rong J., Wang Q. (2016). Improved Productivity of Neutral Lipids in *Chlorella* sp. A2 by Minimal Nitrogen Supply. Front. Microbiol..

[100] Gómez Cardozo J.R., Beigbeder J.-B., Dantas J.M.D.M., Lavoie J.-M. (2023). High-Gravity Fermentation for Bioethanol Production from Industrial Spent Black Cherry Brine Supplemented with Whey. Fermentation.

[101] Alharbi R.M. (2024). Urea-N Influences Biomass Yield, Neutral Lipids Accumulation, and Unsaturated Fatty Acid Production in Photoautotrophically Grown Microalga *Chlorella* sp.. Biocatal. Agric. Biotechnol..

[102] Sigurdarson J.J., Svane S., Karring H. (2020). Development of a M9-based Urea Medium (M9U) for Sensitive and Real-time Monitoring of Ureolytic Activity of Bacteria and Cell-free Urease. MicrobiologyOpen.

[103] Salami S.A., Moran C.A., Warren H.E., Taylor-Pickard J. (2020). A Meta-Analysis of the Effects of Slow-Release Urea Supplementation on the Performance of Beef Cattle. Animals.

[104] Salami S.A., Moran C.A., Warren H.E., Taylor-Pickard J. (2021). Meta-Analysis and Sustainability of Feeding Slow-Release Urea in Dairy Production. PLoS ONE.

[105] Abdullah E.Y., Ali H.T., Ahmet O.G. (2021). Decarbonization in Ammonia Production, New Technological Methods in Industrial Scale Ammonia Production and Critical Evaluations. Heliyon.

[106] Bora N., Kumar Singh A., Pal P., Kumar Sahoo U., Seth D., Rathore D., Bhadra S., Sevda S., Venkatramanan V., Prasad S. (2024). Green Ammonia Production: Process Technologies and Challenges. Fuel.

[107] Brondi M., Eisa M., Bortoletto-Santos R., Drapanauskaite D., Reddington T., Williams C., Ribeiro C., Baltrusaitis J. (2023). Recovering, Stabilizing, and Reusing Nitrogen and Carbon from Nutrient-Containing Liquid Waste as Ammonium Carbonate Fertilizer. Agriculture.

[108] Xu D., Zhong B., Wang X., Li X., Zhong Y., Yan Z., Yang J., Li X., Wang Y., Zhou X. (2022). The Development Road of Ammonium Phosphate Fertilizer in China. Chin. J. Chem. Eng..

[109] Wang C., Walsh S.D.C., Longden T., Palmer G., Lutalo I., Dargaville R. (2023). Optimising Renewable Generation Configurations of Off-Grid Green Ammonia Production Systems Considering Haber-Bosch Flexibility. Energy Convers. Manag..

[110] Kuypers M.M.M., Marchant H.K., Kartal B. (2018). The Microbial Nitrogen-Cycling Network. Nat. Rev. Microbiol..

[111] Liu M., Wang T., Wang L., Xiao H., Li J., Duan C., Gao L., Liu Y., Yan H., Zhang Y. (2024). Core Microbiota for Nutrient Digestion Remained and Ammonia Utilization Increased after Continuous Batch Culture of Rumen Microbiota In Vitro. Front. Microbiol..

[112] Arandia-Gorostidi N., Jaffe A.L., Parada A.E., Kapili B.J., Casciotti K.L., Salcedo R.S.R., Baumas C.M.J., Dekas A.E. (2024). Urea Assimilation and Oxidation Support Activity of Phylogenetically Diverse Microbial Communities of the Dark Ocean. ISME J..

[113] Kemsawasd V., Viana T., Ardö Y., Arneborg N. (2015). Influence of Nitrogen Sources on Growth and Fermentation Performance of Different Wine Yeast Species during Alcoholic Fermentation. Appl. Microbiol. Biotechnol..

[114] Papagianni M., Wayman F., Mattey M. (2005). Fate and Role of Ammonium Ions during Fermentation of Citric Acid by *Aspergillus niger*. Appl. Environ. Microbiol..

[115] Müller T., Walter B., Wirtz A., Burkovski A. (2006). Ammonium Toxicity in Bacteria. Curr. Microbiol..

[116] Li X., Yu F., Liu K., Zhang M., Cheng Y., Wang F., Wang S., Han R., Xue Z. (2022). Uncovering the Effects of Ammonium Sulfate on Neomycin B Biosynthesis in *Streptomyces fradiae* SF-2. Fermentation.

[117] Ardin A.C., Fujita K., Nagayama K., Takashima Y., Nomura R., Nakano K., Ooshima T., Matsumoto-Nakano M. (2014). Identification and Functional Analysis of an Ammonium Transporter in *Streptococcus mutans*. PLoS ONE.

[118] Lee Y.J., Moon B.C., Lee D.K., Ahn J.H., Gong G., Um Y., Lee S.-M., Kim K.H., Ko J.K. (2025). Sustainable Production of Microbial Protein from Carbon Dioxide in the Integrated Bioelectrochemical System Using Recycled Nitrogen Sources. Water Res..

[119] Chen G., Zhao L., Qi Y. (2015). Enhancing the Productivity of Microalgae Cultivated in Wastewater toward Biofuel Production: A Critical Review. Appl. Energy.

[120] Metin U., Altınbaş M. (2024). Evaluating Ammonia Toxicity and Growth Kinetics of Four Different Microalgae Species. Microorganisms.

[121] Scherholz M.L., Curtis W.R. (2013). Achieving pH Control in Microalgal Cultures through Fed-Batch Addition of Stoichiometrically-Balanced Growth Media. BMC Biotechnol..

[122] Popa M.D., Simionov I.-A., Petrea S.M., Georgescu P.-L., Ifrim G.A., Iticescu C. (2025). Efficiency of Microalgae Employment in Nutrient Removal (Nitrogen and Phosphorous) from Municipal Wastewater. Water.

[123] Kundu P., Dutta N., Bhattacharya S. (2024). Application of Microalgae in Wastewater Treatment with Special Reference to Emerging Contaminants: A Step towards Sustainability. Front. Anal. Sci..

[124] Geisseler D., Horwath W.R., Joergensen R.G., Ludwig B. (2010). Pathways of Nitrogen Utilization by Soil Microorganisms–A Review. Soil Biol. Biochem..

[125] Jiang M., Zhao D., Huang L., Zeng Y., Liu J., Xiang H., Zheng Y. (2023). The Role of Glutamine Synthetase in Regulating Ammonium Assimilation and Iron-Only Nitrogenase Expression in a Photosynthetic Diazotroph. Microbiol. Spectr..

[126] Kawade K., Tabeta H., Ferjani A., Hirai M.Y. (2023). The Roles of Functional Amino Acids in Plant Growth and Development. Plant Cell Physiol..

[127] Cai T., Park S.Y., Li Y. (2013). Nutrient Recovery from Wastewater Streams by Microalgae: Status and Prospects. Renew. Sustain. Energy Rev..

[128] Zhu J., Jia Y., Yu G., Wang Q., He N., Chen Z., He H., Zhu X., Li P., Zhang F. (2025). Changing Patterns of Global Nitrogen Deposition Driven by Socio-Economic Development. Nat. Commun..

[129] Liu T., Duan H., Lücker S., Zheng M., Daims H., Yuan Z., Guo J. (2024). Sustainable Wastewater Management through Nitrogen-Cycling Microorganisms. Nat. Water.

[130] Ding S., Jiang L., Hu J., Huang W., Lou L. (2023). Microbiome Data Analysis via Machine Learning Models: Exploring Vital Players to Optimize Kitchen Waste Composting System. Bioresour. Technol..

[131] Mishra S., Singh V., Cheng L., Hussain A., Ormeci B. (2022). Nitrogen Removal from Wastewater: A Comprehensive Review of Biological Nitrogen Removal Processes, Critical Operation Parameters and Bioreactor Design. J. Environ. Chem. Eng..

[132] Selvam A., Ilamathi P.M.K., Udayakumar M., Murugesan K., Banu J.R., Khanna Y., Wong J. (2021). Food Waste Properties. Current Developments in Biotechnology and Bioengineering.

[133] Manu M.K., Li D., Liwen L., Jun Z., Varjani S., Wong J.W.C. (2021). A Review on Nitrogen Dynamics and Mitigation Strategies of Food Waste Digestate Composting. Bioresour. Technol..

[134] Kwan T.H., Hu Y., Lin C.S.K. (2016). Valorisation of Food Waste via Fungal Hydrolysis and Lactic Acid Fermentation with *Lactobacillus casei* Shirota. Bioresour. Technol..

[135] Alrbai M., Al-Dahidi S., Shboul B., Abusorra M., Hayajneh H. (2024). Techno-Economic Feasibility Study of Ammonia Recovery from Sewage Sludge Digestate in Wastewater Treatment Plants. Clean. Environ. Syst..

[136] Nancharaiah Y.V., Kiran Kumar Reddy G. (2018). Aerobic Granular Sludge Technology: Mechanisms of Granulation and Biotechnological Applications. Bioresour. Technol..

[137] Mohammadkhani G., Mahboubi A., Plöhn M., Funk C., Ylitervo P. (2024). Total Ammonia Removal from Anaerobic Digestion Effluents of Municipal Sewage Sludge Using Nordic Microalgae. Algal Res..

[138] Sancho I., Licon E., Valderrama C., De Arespacochaga N., López-Palau S., Cortina J.L. (2017). Recovery of Ammonia from Domestic Wastewater Effluents as Liquid Fertilizers by Integration of Natural Zeolites and Hollow Fibre Membrane Contactors. Sci. Total Environ..

[139] Yu Y., Lei Z., Yuan T., Jiang Y., Chen N., Feng C., Shimizu K., Zhang Z. (2017). Simultaneous Phosphorus and Nitrogen Recovery from Anaerobically Digested Sludge Using a Hybrid System Coupling Hydrothermal Pretreatment with MAP Precipitation. Bioresour. Technol..

[140] Qi B., Jiang X., Wang H., Li J., Zhao Q., Li R., Wang W. (2021). Resource Recovery from Liquid Digestate of Swine Wastewater by an Ultrafiltration Membrane Bioreactor (UF-MBR) and Reverse Osmosis (RO) Process. Environ. Technol. Innov..

[141] Yang J., Zhang J., Du X., Gao T., Cheng Z., Fu W., Wang S. (2024). Ammonia Inhibition in Anaerobic Digestion of Organic Waste: A Review. Int. J. Environ. Sci. Technol..

[142] Mangwe M.C., Mason W.A., Reed C.B., Spaans O.K., Pacheco D., Bryant R.H. (2025). A Systematic Review and Meta-Analysis of Cow-Level Factors Affecting Milk Urea Nitrogen and Urinary Nitrogen Output Under Pasture-Based Diets. J. Dairy Sci..

[143] Sadh P.K., Duhan S., Duhan J.S. (2018). Agro-Industrial Wastes and Their Utilization Using Solid State Fermentation: A Review. Bioresour. Bioprocess..

[144] Sarangi P.K., Vivekanand V., Mohanakrishna G., Pattnaik B., Muddapur U.M., Aminabhavi T.M. (2023). Production of Bioactive Phenolic Compounds from Agricultural By-Products towards Bioeconomic Perspectives. J. Clean. Prod..

[145] Gervasi T., Pellizzeri V., Calabrese G., Di Bella G., Cicero N., Dugo G. (2018). Production of Single Cell Protein (SCP) from Food and Agricultural Waste by Using *Saccharomyces cerevisiae*. Nat. Prod. Res..

[146] Yi Y., Li J., Zhou P., Jia F., Chen Y., Li D. (2024). Production of Single Cell Protein Rich in Potassium by *Nectaromyces Rattus* Using Biogas Slurry and Molasses. J. Environ. Manag..

[147] Yan J., Han B., Gui X., Wang G., Xu L., Yan Y., Madzak C., Pan D., Wang Y., Zha G. (2018). Engineering *Yarrowia lipolytica* to Simultaneously Produce Lipase and Single Cell Protein from Agroindustrial Wastes for Feed. Sci. Rep..

[148] Spalvins K., Zihare L., Blumberga D. (2018). Single Cell Protein Production from Waste Biomass: Comparison of Various Industrial by-Products. Energy Procedia.

[149] Tropea A., Ferracane A., Albergamo A., Potortì A.G., Lo Turco V., Di Bella G. (2022). Single Cell Protein Production through Multi Food-Waste Substrate Fermentation. Fermentation.

[150] Putri D., Ulhidayati A., Musthofa I.A., Wardani A.K. (2018). Single Cell Protein Production of *Chlorella* sp. Using Food Processing Waste as a Cultivation Medium. IOP Conf. Ser. Earth Environ. Sci..

[151] Van Peteghem L., Matassa S., Rabaey K., Sakarika M. (2023). Microbial Protein from Recovered Nitrogen: Nutritional Quality, Safety, and Feasibility Assessment. Sci. Total Environ..

[152] Voutilainen E., Pihlajaniemi V., Parviainen T. (2021). Economic Comparison of Food Protein Production with Single-Cell Organisms from Lignocellulose Side-Streams. Bioresour. Technol. Rep..

[153] Van Peteghem L., Matassa S., Sakarika M. (2025). Fueling the Protein Transition: Can Waste-Derived Ethanol Enable Efficient and High-Quality Microbial Protein Production?. Bioresour. Technol..

[154] Peterson E.C., Siao R., Chua G.G., Busran C.T., Pavlovic R., Thong A., Hermansen C., Sofeo N., Kanagasundaram Y., Weingarten M. (2023). Single Cell Protein and Oil Production from Solid Cocoa Fatty Acid Distillates Co-Fed Ethanol. Bioresour. Technol..

[155] Campos-Valdez A., Kirchmayr M.R., Barrera-Martínez I., Casas-Godoy L. (2023). Sustainable Production of Single-Cell Oil and Protein from Wastepaper Hydrolysate: Identification and Optimization of a *Rhodotorula Mucilaginosa* Strain as a Promising Yeast. FEMS Yeast Res..

[156] Devanthi P.V.P., Pratama F., Pramanda I.T., Bani M.D., Kadar A.D., Kho K. (2024). Exploring the Potential of *Aspergillus oryzae* for Sustainable Mycoprotein Production Using Okara and Soy Whey as Cost-Effective Substrates. J. Fungi.

[157] Santin A., Russo M.T., Ferrante M.I., Balzano S., Orefice I., Sardo A. (2021). Highly Valuable Polyunsaturated Fatty Acids from Microalgae: Strategies to Improve Their Yields and Their Potential Exploitation in Aquaculture. Molecules.

[158] Ritala A., Häkkinen S.T., Toivari M., Wiebe M.G. (2017). Single Cell Protein—State-of-the-Art, Industrial Landscape and Patents 2001–2016. Front. Microbiol..

[159] Batista Meneses D., Montes De Oca-Vásquez G., Vega-Baudrit J.R., Rojas-Álvarez M., Corrales-Castillo J., Murillo-Araya L.C. (2022). Pretreatment Methods of Lignocellulosic Wastes into Value-Added Products: Recent Advances and Possibilities. Biomass Convers. Biorefinery.

[160] He Y., Liu Y., Zhang M. (2024). Hemicellulose and Unlocking Potential for Sustainable Applications in Biomedical, Packaging, and Material Sciences: A Narrative Review. Int. J. Biol. Macromol..

[161] Kavya, Vashisht M., Jain B., Shrivastava S. (2024). Transforming Waste into Wealth: A Review on Microbial Conversion of Organic Municipal Wastes to Value-Added Products. Discov. Environ..

[162] Zhou Z., Zheng X., Hua Y., Guo M., Sun X., Huang Y., Dong L., Yu S. (2024). Enhancing Nitrogen Removal in Combined Sewage Overflows by Using Bio-Fluidized Bed with Ceramic Waste Powder Carriers: Effects and Mechanisms. Environ. Sci. Pollut. Res..

[163] Dutta D., Arya S., Kumar S. (2021). Industrial Wastewater Treatment: Current Trends, Bottlenecks, and Best Practices. Chemosphere.

[164] Jiang P., Zhou T., Bai J., Zhang Y., Li J., Zhou C., Zhou B. (2023). Nitrogen-Containing Wastewater Fuel Cells for Total Nitrogen Removal and Energy Recovery Based on Cl•/ClO• Oxidation of Ammonia Nitrogen. Water Res..

[165] Grenni P., Ancona V., Barra Caracciolo A. (2018). Ecological Effects of Antibiotics on Natural Ecosystems: A Review. Microchem. J..

[166] Rasool K., Hussain S., Shahzad A., Miran W., Mahmoud K.A., Ali N., Almomani F. (2023). Comprehensive Insights into Sustainable Conversion of Agricultural and Food Waste into Microbial Protein for Animal Feed Production. Rev. Environ. Sci. Biotechnol..

[167] Shen M., Song B., Zhou C., Almatrafi E., Hu T., Zeng G., Zhang Y. (2022). Recent Advances in Impacts of Microplastics on Nitrogen Cycling in the Environment: A Review. Sci. Total Environ..

[168] Zhu J., Ren A., Jiao J., Shen W., Yang L., Zhou C., Tan Z. (2022). Effects of Non-Protein Nitrogen Sources on In Vitro Rumen Fermentation Characteristics and Microbial Diversity. Front. Anim. Sci..

[169] Xu X., Hui D., King A.W., Song X., Thornton P.E., Zhang L. (2015). Convergence of Microbial Assimilations of Soil Carbon, Nitrogen, Phosphorus and Sulfur in Terrestrial Ecosystems. Sci. Rep..

[170] Einsle O. (2023). Catalysis and Structure of Nitrogenases. Curr. Opin. Struct. Biol..

[171] Campbell W.H. (1999). NITRATE REDUCTASE STRUCTURE, FUNCTION AND REGULATION: Bridging the Gap between Biochemistry and Physiology. Annu. Rev. Plant Physiol. Plant Mol. Biol..

[172] Neis E., Dejong C., Rensen S. (2015). The Role of Microbial Amino Acid Metabolism in Host Metabolism. Nutrients.

[173] Yelamanchi S.D., Jayaram S., Thomas J.K., Gundimeda S., Khan A.A., Singhal A., Keshava Prasad T.S., Pandey A., Somani B.L., Gowda H. (2016). A Pathway Map of Glutamate Metabolism. J. Cell Commun. Signal..

[174] Brosnan J.T., Brosnan M.E. (2013). Glutamate: A Truly Functional Amino Acid. Amino Acids.

[175] Gao J.-P., Su Y., Jiang S., Liang W., Lou Z., Frugier F., Xu P., Murray J.D. (2024). Applying Conventional and Cell-Type-Specific CRISPR/Cas9 Genome Editing in Legume Plants. aBIOTECH.

[176] Shang Y., Shi H., Liu M., Lan P., Li D., Liu X., Wang M., Zhang Z., Chen S. (2025). Using Synthetic Biology to Express Nitrogenase Biosynthesis Pathway in Rice and to Overcome Barriers of Nitrogenase Instability in Plant Cytosol. Trends Biotechnol..

[177] Joshi R., Sharma V., Kuila A., Kuila A., Sharma V. (2018). Fermentation Technology: Current Status and Future Prospects. Principles and Applications of Fermentation Technology.

[178] Yang X., Yuan L., Zeeshan M., Yang C., Gao W., Zhang G., Wang C. (2025). Optimization of Fermentation Conditions to Increase the Production of Antifungal Metabolites from *Streptomyces* sp. KN37. Microb. Cell Factories.

[179] Pereira A.A., Yaverino-Gutierrez M.A., Monteiro M.C., Souza B.A., Bachheti R.K., Chandel A.K. (2025). Precision Fermentation in the Realm of Microbial Protein Production: State-of-the-Art and Future Insights. Food Res. Int..

[180] Soccol C.R., Costa E.S.F.D., Letti L.A.J., Karp S.G., Woiciechowski A.L., Vandenberghe L.P.D.S. (2017). Recent Developments and Innovations in Solid State Fermentation. Biotechnol. Res. Innov..

[181] Pandey A. (2003). Solid-State Fermentation. Biochem. Eng. J..

[182] Mienda B.S., Idi A., Umar A. (2011). Microbiological Features of Solid State Fermentation and Its Applications. An Overview. Res. Biotechnol..

[183] Betchem G., Monto A.R., Lu F., Billong L.F., Ma H. (2024). Prospects and Application of Solid-State Fermentation in Animal Feed Production-a Review. Ann. Anim. Sci..

[184] Li B., Zhao C., Sun Q., Chen K., Zhao X., Xu L., Yang Z., Peng H. (2023). Effects of Ammonification–Steam Explosion Pretreatment on the Production of True Protein from Rice Straw during Solid-State Fermentation. Sustainability.

[185] Marius K.S., Mahamadi N., Ibrahim K., Iliassou M., Sonagnon H.S.K., Yerobessor D., Wahauwouele H.C., Essodolom T., Alfred S.T. (2018). Production of Single Cell Protein (SCP) and Essentials Amino Acids from *Candida utilis* FMJ12 by Solid State Fermentation Using Mango Waste Supplemented with Nitrogen Sources. Afr. J. Biotechnol..

[186] Maxwell O.I., Chinwuba U.B., Onyebuchukwu M.G. (2019). Protein Enrichment of Potato Peels Using *Saccharomyces Cerevisiae* via Solid-State Fermentation Process. Adv. Chem. Eng. Sci..

[187] Kong S., Wang S., He Y., Wang N., Wang Z., Weng L., Liu D., Zhao X., Chen J., Xu J. (2024). Three-Stage Solid-State Fermentation Technology for Distillers’ Grain Feed Protein Based on Different Microorganisms Considering Oxygen Requirements. Fermentation.

[188] Wang S., Wang Z., Wang N., Wang S., Zeng S., Xu Z., Liu D., Zhao X., Liu F., Xu J. (2025). Efficient Conversion of Corn Straw to Feed Protein through Solid-State Fermentation Using a Thermophilic Microbial Consortium. Waste Manag..

[189] Chen J., Cai Y., Wang Z., Xu Z., Zhuang W., Liu D., Lv Y., Wang S., Xu J., Ying H. (2024). Solid-State Fermentation of Corn Straw Using Synthetic Microbiome to Produce Fermented Feed: The Feed Quality and Conversion Mechanism. Sci. Total Environ..

[190] Chen J., Cai Y., Wang Z., Xu Z., Li J., Ma X., Zhuang W., Liu D., Wang S., Song A. (2023). Construction of a Synthetic Microbial Community Based on Multiomics Linkage Technology and Analysis of the Mechanism of Lignocellulose Degradation. Bioresour. Technol..

[191] Liu J., Wang S., Wang Z., Shen C., Liu D., Shen X., Weng L., He Y., Wang S., Wang J. (2023). Pretreatment of Luzhou Distiller’s Grains for Feed Protein Production Using Crude Enzymes Produced by a Synthetic Microbial Consortium. Bioresour. Technol..

[192] Arora S., Rani R., Ghosh S. (2018). Bioreactors in Solid State Fermentation Technology: Design, Applications and Engineering Aspects. J. Biotechnol..

[193] Durand A. (2003). Bioreactor Designs for Solid State Fermentation. Biochem. Eng. J..

[194] Mattedi A., Sabbi E., Farda B., Djebaili R., Mitra D., Ercole C., Cacchio P., Del Gallo M., Pellegrini M. (2023). Solid-State Fermentation: Applications and Future Perspectives for Biostimulant and Biopesticides Production. Microorganisms.

[195] Sun H., Luan G., Ma Y., Lou W., Chen R., Feng D., Zhang S., Sun J., Lu X. (2023). Engineered Hypermutation Adapts Cyanobacterial Photosynthesis to Combined High Light and High Temperature Stress. Nat. Commun..

[196] Artola A., Font X., Moral-Vico J., Sánchez A. (2024). The Role of Solid-State Fermentation to Transform Existing Waste Treatment Plants Based on Composting and Anaerobic Digestion into Modern Organic Waste-Based Biorefineries, in the Framework of Circular Bioeconomy. Front. Chem. Eng..

[197] Bürck M., Lemes A.C., Egea M.B., Braga A.R.C. (2024). Exploring the Potential and Challenges of Fermentation in Creating Foods: A Spotlight on Microalgae. Fermentation.

[198] Aloo S.O., Park S., Oyinloye T.M., Lee Y.-W., Cho Y.E., Park S.J., Oh D.-H. (2025). Effects of Liquid State vs. Solid State Lactic Fermentation on Drying, Nutritional Composition, Phytochemical Profile, and In Vitro Neuro-Related Bioactivities of Cheungsam Industrial Hempseed (Korean Breed). Food Biosci..

[199] Tian Y., Li J., Meng J., Li J. (2023). High-Yield Production of Single-Cell Protein from Starch Processing Wastewater Using Co-Cultivation of Yeasts. Bioresour. Technol..

[200] Zhang B., Ren D., Liu Q., Liu X., Bao J. (2023). Coproduction of Single Cell Protein and Lipid from Lignocellulose Derived Carbohydrates and Inorganic Ammonia Salt with Soluble Ammonia Recycling. Bioresour. Technol..

[201] Clement P.N., Nwokoro O. (2019). Production of Single Cell Protein from Hydrolyzed Pineapple (*Ananas comosus*) Peel Using Fungi. Bio-Research.

[202] Cerrone F., O’Connor K.E. (2025). Cultivation of Filamentous Fungi in Airlift Bioreactors: Advantages and Disadvantages. Appl. Microbiol. Biotechnol..

[203] Bakratsas G., Polydera A., Nilson O., Chatzikonstantinou A.V., Xiros C., Katapodis P., Stamatis H. (2023). Mycoprotein Production by Submerged Fermentation of the Edible Mushroom *Pleurotus ostreatus* in a Batch Stirred Tank Bioreactor Using Agro-Industrial Hydrolysate. Foods.

[204] Niyigaba T., Küçükgöz K., Kołożyn-Krajewska D., Królikowski T., Trząskowska M. (2025). Advances in Fermentation Technology: A Focus on Health and Safety. Appl. Sci..

[205] Marcellin E., Angenent L.T., Nielsen L.K., Molitor B. (2022). Recycling Carbon for Sustainable Protein Production Using Gas Fermentation. Curr. Opin. Biotechnol..

[206] Woern C., Grossmann L. (2023). Microbial Gas Fermentation Technology for Sustainable Food Protein Production. Biotechnol. Adv..

[207] Raziq A. (2020). Single Cell Protein (SCP) Production and Potential Substrates: A Comprehensive Review. Pure Appl. Biol..

[208] Vlaeminck E., Uitterhaegen E., Quataert K., Delmulle T., Kontovas S.-S., Misailidis N., Ferreira R.G., Petrides D., De Winter K., Soetaert W.K. (2023). Single-Cell Protein Production from Industrial Off-Gas through Acetate: Techno-Economic Analysis for a Coupled Fermentation Approach. Fermentation.

[209] Wang J., Chen L., Xu J., Ma S., Liang X., Wei Z., Li D., Xue M. (2023). C1 Gas Protein: A Potential Protein Substitute for Advancing Aquaculture Sustainability. Rev. Aquac..

[210] Xu J., Wang J., Ma C., Wei Z., Zhai Y., Tian N., Zhu Z., Xue M., Li D. (2023). Embracing a Low-Carbon Future by the Production and Marketing of C1 Gas Protein. Biotechnol. Adv..

[211] Jain S., Heffernan J., Joshi J., Watts T., Marcellin E., Greening C. (2023). Microbial Conversion of Waste Gases into Single-Cell Protein. Microbiol. Aust..

[212] Li S., Zuo X., Carpenter M.D., Verduzco R., Ajo-Franklin C.M. (2024). Microbial Bioelectronic Sensors for Environmental Monitoring. Nat. Rev. Bioeng..

[213] Kholif A.E., Anele A., Anele U.Y. (2024). Microbial Feed Additives in Ruminant Feeding. AIMS Microbiol..

[214] Zeng W., Guo C., Xu S., Chen J., Zhou J. (2020). High-Throughput Screening Technology in Industrial Biotechnology. Trends Biotechnol..

[215] Zhou P., Gao C., Song W., Wei W., Wu J., Liu L., Chen X. (2024). Engineering Status of Protein for Improving Microbial Cell Factories. Biotechnol. Adv..

[216] Cao K., Cui Y., Sun F., Zhang H., Fan J., Ge B., Cao Y., Wang X., Zhu X., Wei Z. (2023). Metabolic Engineering and Synthetic Biology Strategies for Producing High-Value Natural Pigments in Microalgae. Biotechnol. Adv..

[217] Onn S.M., Koh G.J., Yap W.H., Teoh M.-L., Low C.-F., Goh B.-H. (2024). Recent Advances in Genetic Engineering of Microalgae: Bioengineering Strategies, Regulatory Challenges and Future Perspectives. J. Appl. Phycol..

[218] Grossmann M., Kießling F., Singer J., Schoeman H., Schröder M.-B., Von Wallbrunn C. (2011). Genetically Modified Wine Yeasts and Risk Assessment Studies Covering Different Steps within the Wine Making Process. Ann. Microbiol..

[219] Yang P., Condrich A., Lu L., Scranton S., Hebner C., Sheykhhasan M., Ali M.A. (2024). Genetic Engineering in Bacteria, Fungi, and Oomycetes, Taking Advantage of CRISPR. DNA.

[220] Zimmermann A., Prieto-Vivas J.E., Voordeckers K., Bi C., Verstrepen K.J. (2024). Mutagenesis Techniques for Evolutionary Engineering of Microbes-Exploiting CRISPR-Cas, Oligonucleotides, Recombinases, and Polymerases. Trends Microbiol..

[221] Bleisch R., Freitag L., Ihadjadene Y., Sprenger U., Steingröwer J., Walther T., Krujatz F. (2022). Strain Development in Microalgal Biotechnology-Random Mutagenesis Techniques. Life.

[222] Zhu Z., Ding X., Rang J., Xia L. (2024). Application and Research Progress of ARTP Mutagenesis in Actinomycetes Breeding. Gene.

[223] Liu Y., Chen X., Wei D., Xing X. (2024). Rapid Screening of High-Protein Auxenochlorella Pyrenoidosa Mutant by an Integrated System of Atmospheric and Room Temperature Plasma Mutagenesis and High-Throughput Microbial Microdroplet Culture. Algal Res..

[224] Pan J., Zhang J., Wei H., Liu Q., Xu W., Bao Y. (2024). Optimizing Mycelial Protein Yield in *Pleurotus Djamor* via ARTP Mutagenesis and Hybridization Strategies. J. Biotechnol..

[225] Liu Y., Wang B., Zhang X., Men P., Gu M., Zhou Y., Hu W., Wang Z., Wang M., Huang X. (2024). Improving the Production of Micafungin Precursor FR901379 in *Coleophoma Empetri* Using Heavy-Ion Irradiation and Its Mechanism Analysis. Mycology.

[226] Vasina M., Velecky J., Planas I.J., Marques S.M., Skarupova J., Damborsky J., Bednar D., Mazurenko S., Prokop Z. (2022). Tools for Computational Design and High-Throughput Screening of Therapeutic Enzymes. Adv. Drug Deliv. Rev..

[227] Mavrommati M., Daskalaki A., Papanikolaou S., Aggelis G. (2022). Adaptive Laboratory Evolution Principles and Applications in Industrial Biotechnology. Biotechnol. Adv..

[228] Barrick J.E., Lenski R.E. (2013). Genome Dynamics during Experimental Evolution. Nat. Rev. Genet..

[229] Loewe L., Hill W.G. (2010). The Population Genetics of Mutations: Good, Bad and Indifferent. Philos. Trans. R. Soc. B.

[230] Sun X.-M., Ren L.-J., Ji X.-J., Chen S.-L., Guo D.-S., Huang H. (2016). Adaptive Evolution of *Schizochytrium* sp. by Continuous High Oxygen Stimulations to Enhance Docosahexaenoic Acid Synthesis. Bioresour. Technol..

[231] Konstantinidis D., Pereira F., Geissen E., Grkovska K., Kafkia E., Jouhten P., Kim Y., Devendran S., Zimmermann M., Patil K.R. (2021). Adaptive Laboratory Evolution of Microbial Co-cultures for Improved Metabolite Secretion. Mol. Syst. Biol..

[232] Blasche S., Kim Y., Mars R.A.T., Machado D., Maansson M., Kafkia E., Milanese A., Zeller G., Teusink B., Nielsen J. (2021). Metabolic Cooperation and Spatiotemporal Niche Partitioning in a Kefir Microbial Community. Nat. Microbiol..

[233] Ding X., Yang W., Du X., Chen N., Xu Q., Wei M., Zhang C. (2023). High-Level and -Yield Production of L-Leucine in Engineered *Escherichia coli* by Multistep Metabolic Engineering. Metab. Eng..

[234] Choe D., Lee J.H., Yoo M., Hwang S., Sung B.H., Cho S., Palsson B., Kim S.C., Cho B.-K. (2019). Adaptive Laboratory Evolution of a Genome-Reduced *Escherichia coli*. Nat. Commun..

[235] LaCroix R.A., Sandberg T.E., O’Brien E.J., Utrilla J., Ebrahim A., Guzman G.I., Szubin R., Palsson B.O., Feist A.M. (2015). Use of Adaptive Laboratory Evolution To Discover Key Mutations Enabling Rapid Growth of *Escherichia coli* K-12 MG1655 on Glucose Minimal Medium. Appl. Environ. Microbiol..

[236] Meng X., Hu G., Li X., Gao C., Song W., Wei W., Wu J., Liu L. (2025). A Synthetic Methylotroph Achieves Accelerated Cell Growth by Alleviating Transcription-Replication Conflicts. Nat. Commun..

